# Hybrid Organic–Inorganic–Organic Isoporous Membranes with Tunable Pore Sizes and Functionalities for Molecular Separation

**DOI:** 10.1002/adma.202105251

**Published:** 2021-09-27

**Authors:** Zhenzhen Zhang, Assaf Simon, Clarissa Abetz, Martin Held, Anke‐Lisa Höhme, Erik S. Schneider, Tamar Segal‐Peretz, Volker Abetz

**Affiliations:** ^1^ Helmholtz‐Zentrum Hereon Institute of Membrane Research Max‐Planck‐Str. 1 21502 Geesthacht Germany; ^2^ Department of Chemical Engineering Technion‐ Israel Institute of Technology Haifa 3200003 Israel; ^3^ Universität Hamburg Institute of Physical Chemistry Martin‐Luther‐King‐Platz 6 20146 Hamburg Germany

**Keywords:** atomic layer deposition, block copolymer membranes, metal oxide, nanochannels, silanization

## Abstract

Accomplishing on‐demand molecular separation with a high selectivity and good permeability is very desirable for pollutant removal and chemical and pharmaceutical processing. The major challenge for sub‐10 nm filtration of particles and molecules is the fabrication of high‐performance membranes with tunable pore size and designed functionality. Here, a versatile top‐down approach is demonstrated to produce such a membrane using isoporous block copolymer membranes with well‐defined pore sizes combined with growth of metal oxide using sequential infiltration synthesis and atomic layer deposition (SIS and ALD). The pore size of the membranes is tuned by controlled metal oxide growth within and onto the polymer channels, enabling up to twofold pore diameter reduction. Following the growth, the distinct functionalities are readily incorporated along the membrane nanochannels with either hydrophobic, cationic, or anionic groups via straightforward and scalable gas/liquid–solid interface reactions. The hydrophilicity/hydrophobicity of the membrane nanochannel is significantly changed by the introduction of hydrophilic metal oxide and hydrophobic fluorinated groups. The functionalized membranes exhibit a superior selectivity and permeability in separating 1–2 nm organic molecules and fractionating similar‐sized proteins based on size, charge, and hydrophobicity. This demonstrates the great potential of organic–inorganic–organic isoporous membranes for high‐performance molecular separation in numerous applications.

## Graphical abstract

Novel hybrid organic–inorganic–organic isoporous membranes are fabricated via sequential infiltration synthesis and atomic layer deposition within block copolymer membranes followed by addition of functional organic molecules. The membrane pore sizes are controlled by metal oxide growth in a predictive manner. The hybrid membranes exhibit superior selectivity and permeability in separating 1–2 nm organic molecules and similar‐sized proteins.

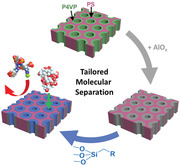

## 1 Introduction

Membrane‐based separation offers a scalable, green, and energy‐efficient tool for numerous applications, including chemical, biochemical, and pharmaceutical processing, potable water purification, and wastewater treatment.^[^ ^1^ ^]^ Central to membrane performance is the ability to fabricate by design the pore channel at the nanoscale level and the pore functionality.^[^ ^2^ ^]^ When targeting molecular separation, the overarching goal of this design is to achieve high‐performance membrane system through tunable pore size with a narrow pore size distribution and designed pore functionality for specific molecular separation.

Pore uniformity is a prerequisite for regulating the selective transport of target molecules. Several techniques are available nowadays for fabricating surfaces with well‐ordered uniform pores of nano‐ to micrometer size, such as anodizing, track‐etching, breath‐figure assembly, and block copolymer (BCP) self‐assembly. Breath‐figure assembly is based on templating ordered membranes using condensed water droplets. It is a captivating route for microsized isoporous membranes due to its simplicity and cost effectiveness.^[^ ^3^ ^]^ The surface within breath figures can be functionalized by decorating it with self‐assembled nanoparticles.^[^ ^4^ ^]^ Macroporous membranes with isoporous surface in flat sheet or hollow fiber geometry can be realized by alternatives to breath‐figure assembly in a nonintermittent manner.^[^ ^5^ ^]^ Anodic aluminum oxide (AAO) membranes are thermally stable, inorganic, nanosized isoporous membranes with highly aligned cylindrical pores through thickness of tens of micrometers.^[^ ^6^ ^]^ Polymeric nanosized isoporous membranes, on the other hand, are easier to scale up and have better mechanical robustness than AAO. Therefore, they are considered as attractive platforms for controlling both pores’ size and pore functionalities for molecular separation.

Several approaches have been demonstrated in polymeric membrane fabrication for molecular separation, mainly based on polycarbonate track‐etched (PCTE) membranes, including electroless deposition of gold nanotubes followed by chemisorption of functionalized thiols, initiated chemical vapor deposition, and self‐assembled polyelectrolyte deposition.^[^ ^7^ ^]^ In contrast to track‐etched membranes, BCP membranes possess both high porosity and narrow pore size distribution due to BCP self‐assembly into well‐defined nanostructures.^[^ ^8^ ^]^ A fast, one‐step, and scalable method to fabricate such BCP membranes employs solvent evaporation induced self‐assembly together with nonsolvent induced phase separation (SNIPS).^[^ ^9^ ^]^ A typical SNIPS membrane possesses a rather thin (<200 nm) top selective layer with high density of uniform pores (>10^14^ pores m^−2^) above a highly porous, mechanically robust supporting sublayer.^[^ ^10^ ^]^ Such unique integral asymmetric isoporous structure can yield high permeance while ensuring good selectivity.^[^ ^11^ ^]^ Most fabricated BCP membranes, however, are limited by pore size to the ultrafiltration regime (10–100 nm) and lack desired pore functionality to achieve molecular separations. Recently, we reported a bottom‐up design of BCP molecular structure to create polymer nanochannels in the nanofiltration regime for molecular separations. The pore size and pore functionality tuning was achieved by one‐step postfunctionalization.^[^ ^12^ ^]^ However, systematic variation of pore size and function can be only realized by separate control over each of them. It remains a challenge to establish a versatile method to independently tune the pore size and the pore functionality with more freedom and flexibility for on‐demand molecular separations.

Atomic layer deposition (ALD) of inorganic materials offers a facile and robust top‐down approach for modifying and engineering membrane surfaces due to its ability to grow thin inorganic coatings on the walls of the tortuous porous network with sub‐nanometer thickness precision.^[^ ^13^ ^]^ ALD has been shown to fine‐tune membranes’ pore size,^[^ ^10^, ^14^ ^]^ introduce hydrophobicity,^[^ ^15^ ^]^ and enhance antifouling properties^[^ ^16^ ^]^ and thermal and electrochemical stability.^[^ ^17^ ^]^

A recent extension of ALD, named sequential infiltration synthesis (SIS), enables to go beyond thin conformal coating and grow inorganic materials within the polymer volume. In SIS, high precursor partial pressures and long diffusion times lead to precursor sorption and diffusion within the polymers.^[^ ^18^ ^]^ Favorable interactions between precursors and polar polymer moieties yields selective growth of inorganic materials within the polar blocks of BCP,^[^ ^19^ ^]^ enabling fabrication of hybrid organic–inorganic materials and well‐defined inorganic nanostructures templated by BCPs.^[^ ^20^ ^]^ Common BCP blocks containing carbonyl^[^ ^21^ ^]^ or pyridine moieties^[^ ^18^, ^22^ ^]^ are perfectly suited for such a selective growth by SIS.

**Figure 1:**
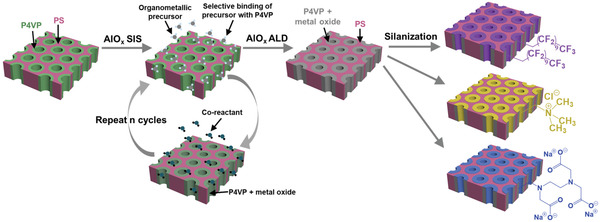
Schematic illustration of the fabrication process of hybrid organic–inorganic–organic isoporous membranes. Polystyrene‐*block*‐poly(4‐vinylpyridine) (PS‐*b*‐P4VP) membrane is modified by SIS with selectively binding an organometallic precursor, followed by exposure to a co‐reactant precursor, resulting in growth of AlO_x_ within the P4VP domains. To further reduce the pore size, AlO_x_ ALD is performed. The AlO_x_‐treated membrane is then postfunctionalized via silanization to introduce different functionalities, i.e., fluorinated groups and cationic and anionic groups.

Herein, we present novel hybrid organic–inorganic–organic isoporous membranes via AlO_x_ SIS and ALD within asymmetric BCP membranes followed by addition of functional organic molecules. With SIS, we selectively swell the pore channel polymer block and create a hybrid polymer–inorganic interface that facilitates further pore size tuning by ALD. Notably, the new inorganic pore channel surface provides a basis for incorporating distinct functional groups with hydrophobicity, cationic, and anionic charge via mild, one‐step, and scalable silanization reactions (**Figure** **1**). The resulting hybrid organic–inorganic–organic isoporous membranes enable highly efficient separation of small molecules with 1–2 nm lateral dimensions and similarly sized biomolecules based on size, charge, and hydrophobicity.

## 2 Results and Discussion

**Figure 2:**
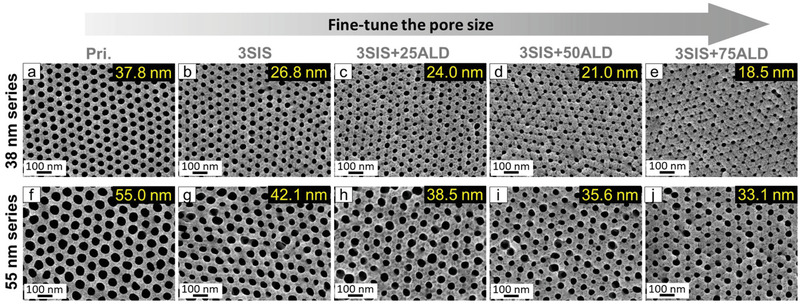
Effect of AlO_x_ growth on the membrane pore size: two series of membranes with initial pore size of a–e) 38 nm and f–j) 55 nm. SEM images of: a,f) pristine membranes Pri.38 and Pri.55, b,g) modified membranes with three cycles of AlO_x_ SIS (3SIS), additional modifications with c,h) 25 cycles of AlO_x_ ALD (3SIS+25ALD), d,i) 50 cycles of AlO_x_ ALD (3SIS+50ALD), and e,j) 75 cycles of AlO_x_ ALD (3SIS+75ALD).

To evaluate the capabilities of tuning isoporous membranes pore size by AlO_x_ SIS and ALD, we synthesized two polystyrene‐*block*‐poly(4‐vinylpyridine) (PS‐*b*‐P4VP) diblock copolymers with distinct molecular characteristics by living anionic polymerization (Table S1, Supporting Information). Integral asymmetric isoporous membranes with well‐defined pore diameter of 38 nm and 55 nm were prepared, respectively (**Figure** **2**a,f). AlO_x_ SIS and additional ALD deposition were performed on these two membranes to investigate the influence of AlO_x_ growth on the pore diameter (Figure 2; Figure S1, Supporting Information). Interestingly, with merely 3 cycles of AlO_x_ SIS (3SIS), the pore size was pronouncedly reduced (≈12 nm reduction; Figure 2b,g) compared to that of the pristine membranes (Pri.38 and Pri.55). This significant reduction is due to the exclusive infiltration of AlO_x_ into the P4VP pore‐forming block, but not the PS matrix block during the SIS process, leading to P4VP domain swelling within the pore and consequently reducing the pore size.

Additional AlO_x_ growth of 25, 50, and 75 ALD cycles (25ALD, 50ALD, and 75ALD) resulted in pore size reduction with a linear trend for both series of membranes, with an average pore diameter reduction of ≈∼0.12 nm per ALD cycle (Figure 2c–e,h–j; Figure S1, Supporting Information). AlO_x_ growth rate was ≈0.6 Å per cycle, deviating from the measured value of 1 Å per cycle on silicon surfaces. A similar discrepancy was also observed for other reported AlO_x_ growth on high aspect ratio structures such as mesoporous inorganic and organic membranes.^[^ ^14^, ^16^ ^]^ As expected, energy dispersive X‐ray spectroscopy (EDX) results show that the intensity of the Al K‐shell electron peak (≈1.49 eV) increases with an increasing number of ALD cycles (Figure S1, Supporting Information).

It is important to note that the initial 3 cycles of AlO_x_ SIS prior to the ALD cycles played a significant role in the AlO_x_ growth and pore size reduction. When a comparable number of ALD cycles was employed without prior SIS growth, the amount of AlO_x_ was significantly lower than that of 3SIS (Figures S1 and S2b, Supporting Information). This indicates that the initial SIS treatment formed a hybrid AlO_x_–P4VP interface that enhanced the subsequent growth of AlO_x_ during ALD cycles. In addition, SIS is more efficient in reducing the pore size of pristine BCP membranes than ALD (see the Supporting Information for additional details); pore diameter reduction of ≈12 nm was achieved with 3 SIS cycles while 10 cycles of ALD resulted in only mild decrease of 3–4 nm (Figure 2; Figures S1 and S2a, Supporting Information).

Overall, we observed a comparable pore size reduction of ≈20 nm for both series with 3SIS plus 75ALD, independent of the molecular characteristic of PS‐*b*‐P4VP (Figure 2). In the 38 nm membrane, this translated to two‐fold reduction in pore size—reaching sub‐20 nm pores. These results highlight how SIS and ALD can be used to tailor the pore size of isoporous BCP membranes, in a predictive and controlled manner, to obtain the desired pore size.

Pore size tuning of isoporous BCP membranes can be realized by varying the molecular weight of the BCP and within a limited range also by varying the content of pore‐forming block.^[^ ^23^ ^]^ Sub‐10 nm isoporous membranes can be fabricated using BCP with a high Flory–Huggins interaction parameter.^[^ ^24^ ^]^ However, the low molecular weight necessary for the small pores results in poorer mechanical properties due to the lack of polymer chain entanglements. Therefore, it is desired to employ BCP with sufficiently high molecular weight as the starting membrane material. Relying on the established relationship between SIS and ALD cycles and pore size reduction, we can readily fabricate the membrane with a predetermined pore size and better mechanical properties.

The AlO_x_ distribution across the membrane can significantly affect the membrane performance.^[^ ^16b^ ^]^ For example, Asatekin and Gleason showed that the pore geometry (cylindrical vs bottleneck) has a crucial influence on the membrane separation performance, i.e., cylindrical pores provide higher selectivity than bottleneck‐shaped pores with a comparable diameter.^[^ ^7f^ ^]^ To probe the hybrid organic–inorganic PS‐*b*‐P4VP–AlO_x_ pore structure, we performed backscattering electron (BSE) imaging with scanning electron microscopy (SEM) as well as transmission electron microscopy (TEM) and elemental mapping with EDX to visualize the location of AlO_x_.

**Figure 3:**
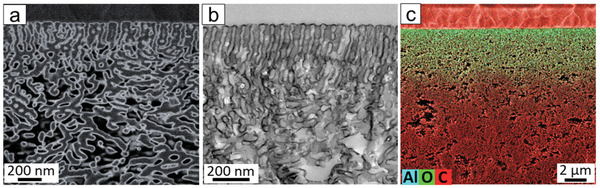
Cross‐sectional images of the 38 nm membrane modified with 3SIS+75ALD (38_3SIS+75ALD): a) backscattered electron image and b) bright‐field TEM image. c) Elemental distribution of carbon, oxygen, and aluminum along the cross‐section of 38_3SIS+75ALD using SEM‐EDX.

Due to the electron density difference between the metal oxide and the polymer matrix, AlO_x_ appears as bright regions in BSE images, whereas it corresponds to the darker regions of TEM images. Top‐down BSE imaging indicates that the AlO_x_ covers the entire membrane top surface, including both P4VP and PS blocks, as can be expected from SIS and ALD processes (Figures S5a–c and S6a–c, Supporting Information). Cross‐sectional BSE and TEM images display a uniform, conformal AlO_x_ layer along the pore wall within the depth of ≈1 µm (**Figure** **3**a,b; Figures S5–S8, Supporting Information). This conformal growth maintains the cylindrical geometry of pore channels while creating a new inorganic channel surface throughout the selective layer of the SNIPS membrane.

The thickness of the AlO_x_ layer was estimated from the TEM images. Both series of 38 and 55 nm membranes showed similar AlO_x_ layer thickness for the same number of SIS and ALD cycles, which confirmed again the versatility of the AlO_x_ growth on PS‐*b*‐P4VP isoporous membranes (Figures S7 and S8 and Table S2, Supporting Information). The elemental distribution of C, O, and Al along the cross‐section was determined by EDX. Interestingly, the maximum penetration depth of AlO_x_ was around 4–5 µm, independent of the membrane pore size (Figure 3c; Figures S9–S17, Supporting Information). The conformal AlO_x_ growth at the top 4–5 µm of the membrane creates a uniform channel surface, which can be used for further modifications.

To probe how the pore size reduction is translated to the molecular weight cut‐off (MWCO), we analyzed the retention tests of poly(ethylene oxide) (PEO) with different molecular weights. The hybrid membranes—38_3SIS+75ALD and 55_3SIS+75ALD showed a sharper MWCO curve and lower MWCO (359 kDa PEO), compared to the pristine membranes—Pri.38 and Pri.55 (1015 kDa PEO; Figure S18, Supporting Information), indicating high uniformity and lack of defects of the AlO_x_ growth.

In addition, the thermal stability of the BCP membranes was enhanced by the SIS and ALD treatments, e.g., the pristine BCP membranes showed stability at 100 °C, whereas 3SIS+75ALD was stable up to 115 °C. This enhanced stability is attributed to the AlO_x_ growth within and onto the P4VP domains via SIS and ALD throughout the top 4–5 µm of the membrane (Figures S19–S25, Supporting Information). Thus, SIS and ALD provide a promising top‐down approach to fabricate thermally stable hybrid organic–inorganic isoporous membranes.

**Figure 4:**
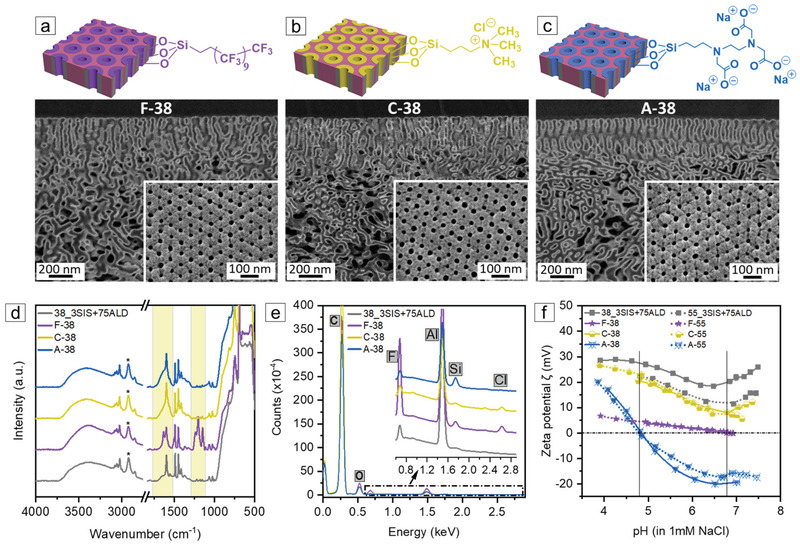
Schematic representation and SEM images of a) F‐38 (fluorinated 38_3SIS+75ALD), b) C‐38 (cationic functionalized 38_3SIS+75ALD), and c) A‐38 (anionic functionalized 38_3SIS+75ALD). d) ATR‐FTIR spectra of 38_3SIS+75ALD, F‐38, C‐38, and A‐38. The relative intensities were normalized using the characteristic CH_2_ stretching vibration (*) of the unreactive PS‐*b*‐P4VP backbone around 2924 cm^−1^. e) Determination of the characteristic elements fluorine (F), silicon (Si), and chlorine (Cl) by EDX along the cross‐section. f) Comparison of the surface zeta potential of two series of 3SIS+75ALD membranes before and after silanization as a function of pH.

In molecular separation, the membrane performance is governed by two key features—the membrane pore size and pores’ surface properties. After we established a method to precisely tune the pore size via SIS and ALD, we turn to control the pores’ surface properties. In the hybrid organic–inorganic membranes, the hydroxyl groups on the AlO_x_ pores’ surface offer new functionalization opportunities through reaction with suitable molecules. This is demonstrated by postfunctionalization of the 3SIS+75ALD modified membranes using various silane coupling agents, i.e., perfluorododecyltrichlorosilane (FDTS), *N*‐[3‐(trimethoxysilyl)propyl]‐*N*,*N*,*N*‐trimethylammonium chloride (TMS‐TMAC), and *N*‐[(3‐trimethoxysilyl)propyl]ethylenediamine triacetic acid trisodium salt (TMS‐EDTA), to obtain hydrophobic, cationic and anionic pore channels, respectively (**Figure** **4a**–c). The reactions were performed in a straightforward, one‐step, and scalable gas/liquid–solid interface reaction.

In the Fourier‐transfrom infrared (FTIR) spectra (Figure 4d; Figure S26d, Supporting Information), the FDTS‐treated 3SIS+75ALD membranes (F‐38 and F‐55) show new characteristic vibrations at 1150, 1204, and 1238 cm^−1^ (between 1100 and 1350 cm^−1^), assigned to the vibration of C–F, in agreement with the new elements—silicon (Si) and fluorine (F) that appear in the corresponding EDX spectra (Figure 4e; Figure S26e, Supporting Information). These results confirm the successful covalent attachment of fluorinated groups on the membranes. In the case of the TMS‐TMAC‐treated 3SIS+75ALD membranes (C–38 and C–55), the FTIR spectra display a broader peak at ≈1600 cm^−1^ compared to that of 38_3SIS+75ALD and 55_3SIS+75ALD, which is attributed to the overlap of the stretching vibrations of quaternary ammonium groups (C–N^+^) with C=N and C=C of the aromatic rings in PS‐*b*‐P4VP (Figure 4d; Figure S26d, Supporting Information).^[^ ^25^ ^]^ Moreover, the presence of characteristic Si and chlorine (Cl) elements is confirmed by the corresponding EDX spectra (Figure 4e; Figure S26e, Supporting Information). The FTIR and EDX analysis therefore confirm that the desired quaternary ammonium moiety was indeed introduced to the membrane by the silanization with TMS‐TMAC.

The FTIR spectra of the TMS‐EDTA‐treated 3SIS+75ALD membranes (A‐38 and A‐55) exhibit new characteristic vibrations as a shoulder peak at ≈1610 cm^−1^ and a broad peak at ≈1400 cm^−1^, assigned to the asymmetric and symmetric stretching vibrations of carboxylate groups (COO^−^) (Figure 4d; Figure S26d, Supporting Information).^[^ ^26^ ^]^ Together with the presence of Si in the corresponding EDX spectra (Figure 4e; Figure S26e, Supporting Information), it clearly shows the successful attachment of the anionic group (COO^−^) on the membrane. SEM images illustrate that all postfunctionalized membranes (i.e., F‐38, F‐55, C‐38, C‐55, A‐38, and A‐55) retain their initial asymmetric isoporous morphology and so does the grown AlO_x_ layer of 38_3SIS+75ALD and 55_3SIS+75ALD (Figure 4a–c; Figures S28 and S29, Supporting Information). Due to the covalent attachment of the functional moieties, all the functionalized membranes show slightly smaller surface pore sizes compared to those of the initial blank 3SIS+75ALD membrane (Table S3, Supporting Information).

The surface zeta potential (ζ) of 38_3SIS+75ALD and 55_3SIS+75ALD is positive at the measured pH range of 4–7.5 (Figure 4f), in good agreement with a previous study.^[^ ^27^ ^]^ F‐38 and F‐55 exhibit a slightly negative ζ at pH >6.77 that becomes slightly positive below pH 6.77, due to coverage of the membrane surface with fluorinated groups. After the introduction of cationic groups, C‐38 and C‐55 still possess a positive ζ in the pH range of 4–7.5. A‐38 and A‐55 display an isoelectric point (IEP) at ≈4.78 and a plateau of highly negative ζ in the pH range of 6–7.5, which is attributed to the presence of the acidic groups (i.e., carboxylic acid) and their corresponding dissociation.^[^ ^12^, ^28^ ^]^

**Figure 5:**
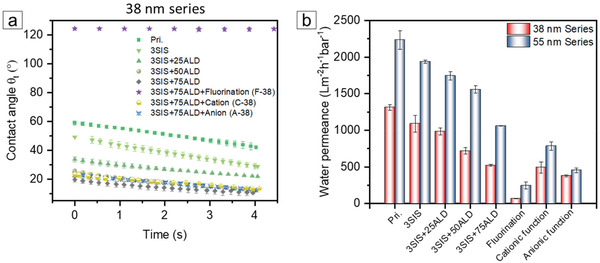
a) The change of the water contact angle on the membrane surface of the 38 nm series as a function of time. b) Water permeance of membranes with the SIS/ALD treatments and different silanizations.

The hydrophilicity/hydrophobicity of the two series of membranes with the SIS and ALD treatments and different silanizations is demonstrated in **Figure** **5**a and Figure S31 in the Supporting Information. The water contact angle θ of the two series of 38 and 55 nm membranes decreases with the increasing number of ALD cycles. With the growth of hydrophilic AlO_x_, the membranes become more hydrophilic than the pristine membranes—Pri.38 and Pri.55 (details are provided in the Supporting Information). Interestingly, the sinking rate of a water droplet through all membranes is similar and does not change with pore size (Figure 5a; Figure S31, Supporting Information). After the incorporation of long fluorinated alkyl groups along the AlO_x_ layer, the hydrophilic membrane turns into a highly hydrophobic membrane with a contact angle of ≈125° (F‐38 in Figure 5a and F‐55 in Figure S31 in the Supporting Information). When introducing the cationic and anionic groups onto the AlO_x_ layer, we observed a slightly more hydrophobic surface compared to 38_3SIS+75ALD and 55_3SIS+75ALD. The density of the attached charged groups seems to be less than that of the hydroxyl groups of the AlO_x_ layer due to the steric hindrance of the bulky silane coupling agents, leading to a lower affinity of water.

The water permeance of the two series of 38 and 55 nm membranes displays a gradual decrease with an increasing number of cycles of SIS/ALD processes (Figure 5b; Table S3, Supporting Information). This is expected due to the trade‐off between the pore size reduction and water permeance, which is also implied by the effective pore size calculation using the Hagen–Poiseuille equation in Figure S32, Supporting Information. F‐38 and F‐55 exhibit a dramatic decrease of water permeance to 68 and 245 L m^−2^ h^−1^ bar^−1^, respectively, compared to that of 38_3SIS+75ALD and 55_3SIS+75ALD (Figure 5b; Table S3, Supporting Information), due to the high energy barrier of the hydrophobic channel. When introducing the cationic and anionic groups to the AlO_x_ layer, we observed a slight decrease in water permeance, compared to the hybrid membranes prior to silanization, which is attributed to the reduction of pore size (Figures 4 and 5b; Table S3, Supporting Information). As expected from the larger pore size, the water permeance of the 55 nm series exceeded that of the 38 nm series (Figure 5b; Table S3, Supporting Information).

To probe hydrophobicity‐based separation of the fluorinated membrane, we chose tris(bipyridine)ruthenium(II) chloride (Ru) and rose bengal (RB) as model molecules. These two molecules are similar in size (≈1 nm) but Ru is more hydrophobic than RB, although still soluble in water.^[^ ^15^ ^]^ The pH of the aqueous solution of Ru and RB is 6.59 and 6.72 (Table S4, Supporting Information), very close to the IEP of F‐38 (IEP = 6.77). The permeability and selectivity of membranes were investigated via pressure‐driven flow, as it is more relevant to a realistic application than concentration‐driven diffusion.^[^ ^12^, ^25^ ^]^

**Figure 6:**
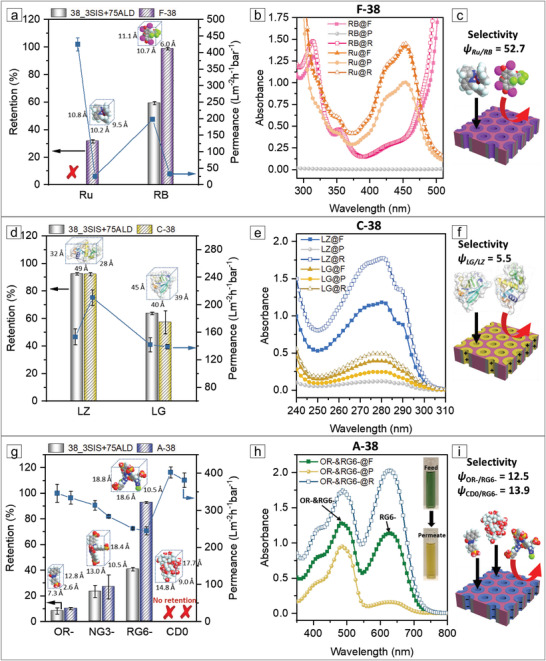
a–c) Hydrophobicity‐based, d–f) charge‐based, and g–i) size/charge‐based selectivity between different organic solutes based on the fluorinated F‐38, cationic 38_3SIS+75ALD and C‐38, and anionic A‐38 membrane, respectively. a,d,g) Retention (column, left *Y*‐axis) and permeance (line+symbol, right *Y*‐axis). b,e,h) Separation behavior determined by UV–vis spectra of the corresponding feed (F), permeate (P), and retentate (R); h) a color change of the feed and permeate solution in the mixed‐solute retention of OR− and RG6−. c,f,i) Schematic representation of separation behavior.

The aqueous solution of Ru freely permeated through 38_3SIS+75ALD without any rejection while 59.2% RB was retained (**Figure** **6**a). This retention is attributed to the adsorption of anionic RB molecules on the positively charged AlO_x_ surface of 38_3SIS+75ALD, forming a negatively charged surface and consequently repelling additional anionic RB molecules, as shown in a previous report.^[^ ^12a^ ^]^ F‐38, on the other hand, retained 31.5% Ru from the aqueous solution compared to 98.7% of the RB molecules (Figure 6a). F‐38 has a negligible positive charge in the pH range of 6.59–6.72 (Figure 4e), leading to a scarce charge impact on the transport of Ru and RB. Thus, the separation behavior of Ru and RB is mainly dictated by the hydrophobicity of the fluorinated membranes. While Ru is hydrophobic, it has an additional energy barrier for entering the highly hydrophobic nanochannels due to its hydration shell, resulting in moderate rejection. RB, on the other hand, is more hydrophilic; even though the highly hydrophobic nanochannels of 15.1 nm in diameter are larger than the size of RB (≈1 nm), such highly hydrophobic nanochannels are sufficiently narrow for hydrophilic RB to exert the strong hydrophilic–hydrophobic repulsion, resulting in high RB rejection. The permeance of Ru and RB solutions through F‐38 is pronouncedly reduced to 25 and 33 L m^−2^ h^−1^ bar^−1^ compared to that through 38_3SIS+75ALD (Figure 6a; Table S6, Supporting Information) due to the hydrophobic nanochannels, as was shown for the permeance of ultrapure water (Figure 5b). Noticeably, the selectivity *Ψ*_Ru/RB_ = 52.7 of F‐38 is 20 times higher compared to *Ψ*_Ru/RB_ = 2.5 of 38_3SIS+75ALD (Table S6, Supporting Information). Importantly, this high selectivity is one order of magnitude higher than a previously reported system of Ru/RB separation (Table S10, Supporting Information).^[^ ^15^ ^]^ These results demonstrate that our organic–inorganic–organic membrane can separate similar sized organic molecules with a lateral dimension of ≈1 nm based on hydrophobicity.

To investigate the separation efficiency of the cationic functionalized membrane, two proteins, similar in size but with different IEP were used as model compounds—lysozyme (LZ, 4.9 nm × 3.2 nm × 2.8 nm, IEP = 11.35) and β‐lactoglobulin (LG, 4.5 nm × 4.0 nm × 3.9 nm, IEP = 5.2–5.3). The pH of the aqueous solution of LZ and LG is around 4.25 and 6.75, respectively. LZ molecules possess a strong overall positive charge while LG has a slightly negative charge (Table S5, Supporting Information). 92.4% and 92.2% of LZ were retained from the aqueous solution by 38_3SIS+75ALD and C‐38, respectively. In both membranes, the positively charged nanochannels are sufficiently narrow to exert a strong electrostatic repulsion on LZ molecules to a similar extent. 38_3SIS+75ALD and C‐38 retained LG with a comparable retention of 63.6% and 57.1%, respectively (Figure 6d), due to the adsorption of negatively charged LG on the positively charged nanochannels and the consequently electrostatic repulsion between LG and the negatively charged nanochannels with adsorbed LG molecules.^[^ ^12a^ ^]^ The permeance of the protein solutions through these membranes is also in a comparable range of 139–153 L m^−2^ h^−1^ bar^−1^, except the LZ solution which passed through C‐38 with a higher permeance of 210 L m^−2^ h^−1^ bar^−1^ (Figure 6d; Table S7, Supporting Information). This higher permeance of LZ solution likely arises from the favorable hydrophobic/hydrophilic interaction between LZ molecules and C‐38. The permeance of the protein solutions is about 5–10 times higher than in previously reported study of protein fractionation.^[^ ^29^ ^]^ As a result, 38_3SIS+75ALD and C‐38 display a comparable selectivity *Ψ*_LG/LZ_ of 4.8 and 5.5, respectively. These results suggest that the positively charged membranes can indeed separate biomolecules (e.g., proteins) of similar size (3–4 nm) through charge‐based selectivity.

To demonstrate the separation performance of anionic functionalized membrane, we probed four organic molecules: three anionic dyes with sulphonate functional groups and different molecular weights (monovalent orange II (OR−), trivalent naphthol green B (NG3−), and hexavalent reactive green 19 (RG6−); 350.32, 878.45, and 1418.93 g mol^−1^, respectively), and a neutral molecule—β‐cyclodextrin (CD0, 1134.98 g mol^−1^). The aqueous solutions of anionic OR−, NG3−, and RG6− permeated through 38_3SIS+75ALD with increasing retention, i.e., 8.2%, 23.4%, and 40.6% (Figure 6g; Table S8, Supporting Information). This trend is attributed to the increasing molecular size and the above mentioned tendency of adsorption between substances with opposite charges (Figure 6a,d).^[^ ^12a^ ^]^ No retention of neutral CD0 was observed due to absence of electrostatic interaction between the CD0 molecules and 38_3SIS+75ALD together with sufficiently large nanochannels (Figure 6g). The pH values of the 0.1 × 10^−3^ m aqueous solutions of OR−, NG3−, RG6−, and CD0 are 6.70, 6.40, 4.82, and 7.06, respectively (Table S4, Supporting Information). Under these pH conditions, the anionic functionalized membrane A‐38 is negatively charged (Figure 4f). In spite of the electrostatic repulsion between charges of equal polarity, the nanochannels of A‐38 are large enough to allow 90% of OR− and 73.1% of NG3− to permeate through the membrane. However, the nanochannels are sufficiently narrow for the larger RG6− to exert a strong electrostatic repulsion, leading to a retention of 92.8%. Moreover, the CD0 solution freely permeated through A‐38 without any rejection (Figure 6g; Table S8, Supporting Information). As a result, the selectivities *Ψ*_OR−/RG6−_, *Ψ*_NG3−/RG6−_, and *Ψ*_CD0/RG6−_ are 12.5, 10.2, and 13.9, respectively. These high selectivities indicate the strong capability of A‐38 to perform charge or size/charge‐based separation of small (1–2 nm) organic molecules. Overall, the permeance of OR−, NG3−, RG6−, and CD0 solutions through this membrane exhibits a reasonable decreasing trend as the retention in the range of 245 to 381 L m^−2^ h^−1^ bar^−1^. Remarkably, the permeance of the RG6− solution through A‐38 was 245 L m^−2^ h^−1^ bar^−1^ while maintaining a high rejection of 92.8%. This high performance is ascribed to distinct uniform functionalized nanochannels with a high number density (Table S8, Supporting Information). Such high permeance is one order of magnitude higher than in typical nanofiltration membranes used for small organic molecules separation.^[^ ^12^, ^30^ ^]^ The selectivity is improved by a factor of two while the permeance is four times higher than a previously reported membrane (Table S10, Supporting Information).^[^ ^12b^ ^]^

To further validate the superior separation performance of the organic–inorganic–organic membranes, the separation of selected model solute mixture was probed. Ru and RB mixture resulted in a turbid suspension due to the electrostatic attraction between them and therefore could not be used (Table S4, Supporting Information). LZ and LG proteins have a similar UV–vis spectroscopic feature (Figure 6e). Therefore, we chose a mixture of OR− and RG6− to permeate through A‐38 as a model system (Figure 6h; Table S9, Supporting Information). A‐38 retained 22.7% OR− and 93.8% RG6− from the mixed aqueous solution with a high permeance of 253 L m^−2^ h^−1^ bar^−1^, obtaining a selectivity *Ψ*_OR−/RG6−_ = 11.7 (Table S9, Supporting Information). The resulting permeance and selectivity are comparable with the values obtained from the single‐solute systems. Unlike the single solute system, OR− and RG6− repelled each other while competing to enter the pores from the mixed solution bulk. Although both had to overcome the energy barrier of electrostatic repulsion to enter the membrane nanochannel, the energy barrier was larger for the hexavalent RG6− than the monovalent OR−. Larger molecular dimensions of RG6− further hindered its entry compared to OR−. Moreover, OR− was likely to change the structural orientation so that the noncharged end enters the nanochannels first, which was not possible for RG6−. Together, these factors resulted in increased retention of OR− and RG6− compared to that of the single solute system, in coherence with a previous study.^[^ ^12a^ ^]^ The high permeance and selectivity demonstrate that the anionic functionalized organic–inorganic–organic membrane exhibits superior separation performance for small organic molecules with a lateral dimension of 1–2 nm, building upon differences in charge and size.

It is interesting to ask whether we can increase the permeance while preserving the desired selectivity by tuning the pore size of the functionalized organic–inorganic–organic membranes. Accordingly, we studied the separation performance of the functionalized 55_3SIS+75ALD membranes, which has an initial larger pore size (Figures S33–S36 and Tables S6–S8, Supporting Information). For all the fluorinated, cationic, and anionic functionalized membranes (F‐55, C‐55, and A‐55), the permeance of model molecules increased with the pore size whereas the corresponding selectivity decreased. This result demonstrates again that both pore size and specific functionality play a vital role in molecular selective transport. It also shows that for targeted molecular separation, the pore size should be tuned to a prerequisite value to exert the specific functionality interaction on the transport of the target molecules. In other words, we can design the membrane to achieve efficient molecular separation for specific target molecules using our degrees of freedom—the asymmetric BCP membrane, SIS and ALD, and fluorinated, cationic, and anionic functionalization.

## 3 Conclusion

We presented novel hybrid organic–inorganic–organic isoporous BCP membranes via AlO_x_ growth with SIS and ALD and organic functionalization. The membrane pore size was tailored by AlO_x_ growth in a wide range, independent of the particular molecular characteristics of BCPs. The AlO_x_ growth with SIS occurs inside the polar pore‐forming P4VP domains with a high conformality and a high penetration depth along the nanochannels. The resulting organic–inorganic membranes exhibit an enhanced thermal stability. By taking advantage of the hydroxyl groups along the AlO_x_ layer, various functional groups, i.e., hydrophobic, cationic, and anionic groups, were readily integrated within the nanochannel, via a straightforward and scalable silanization process. The hydrophilicity or hydrophobicity of the nanochannels was tuned pronouncedly by the AlO_x_ growth or the additional introduction of highly hydrophobic groups, respectively. The retention studies demonstrate the capability of the functionalized organic–inorganic–organic membranes to fractionate biomolecules (e.g., 3–4 nm similar‐sized proteins) and efficiently separate small organic molecules (1–2 nm) from each other with superior selectivity and permeability. The incorporation of inorganic metal oxide into and onto polymeric membranes via SIS and ALD provides a versatile “top‐down” approach to fabricate high‐performance membranes with tunable pore size and functionality to fulfill the on‐demand separation in many fields including chemical and pharmaceutical separations, biomolecules fractionation, and purification.

## Conflict of Interest

The authors declare no conflict of interest.

## Supporting information

Supporting Information

## Data Availability

Research data are not shared.
