# Supplementary material for: Hybrid Organic–Inorganic–Organic Isoporous Membranes with Tunable Pore Sizes and Functionalities for Molecular Separation
Source: Adv Mater. 2021 Sep 27;33(48):2105251. doi: 10.1002/adma.202105251 (PMC11469200; doi:10.1002/adma.202105251)
Supplement: Supplementary file 1 — Supporting Information [file ADMA-33-2105251-s001.pdf]

© 2021 Wiley-VCH GmbH

ADVANCED MATERIALS

Supporting Information

for *Adv. Mater.,* DOI: 10.1002/adma.202105251

Hybrid Organic–Inorganic–Organic Isoporous Membranes with Tunable Pore Sizes and Functionalities for Molecular Separation

Zhenzhen Zhang, Assaf Simon, Clarissa Abetz, Martin Held, Anke-Lisa Höhme, Erik S. Schneider, Tamar Segal-Peretz, and Volker Abetz**

Hybrid Organic-Inorganic-Organic Isoporous Membranes with Tunable Pore Sizes and Functionalities for Molecular Separation

Zhenzhen Zhang[1,$], Assaf Simon[2,$], Clarissa Abetz[1], Martin Held[1], Anke-Lisa Höhme[1], Erik S. Schneider[1], Tamar Segal-Peretz[2], Volker Abetz[1,3*]*

[1]Helmholtz-Zentrum Hereon, Institute of Membrane Research, Max-Planck-Str.1, 21502 Geesthacht, Germany

[2]Department of Chemical Engineering, Technion-Israel Institute of Technology, Haifa-3200003, Israel

[3]Universität Hamburg, Institute of Physical Chemistry, Martin-Luther-King-Platz 6, 20146 Hamburg, Germany

[$] These authors contributed equally to this work

E-mail: tamarps@technion.ac.il, volker.abetz@hereon.de

Keywords: block copolymer membrane, atomic layer deposition, silanization, nanochannel, metal oxide

1. Experimental

1.1. Materials

All materials were used as received without further purification unless described specifically. Styrene was purified from aluminum oxide and subsequently distilled from di-*n*-butyl magnesium (Sigma-Aldrich, USA, 1.0 M solution in heptane). 4-vinylpyridine (4VP) was distilled under reduced pressure after being treated twice with ethylaluminum dichloride (Sigma-Aldrich, USA, 1M in hexane). The solvent used for polymerization was tetrahydrofuran (THF), purified from sodium metal, titrated with *sec*-butyl lithium (*s*-BuLi, Sigma-Aldrich, USA, 1.4 M solution in cyclohexane). Perfluorodecyltrichlorosilane (FDTS) was purchased from Sigma-Aldrich. *N*-[(3-Trimethoxysilyl)propyl]ethylenediamine triacetic acid trisodium salt (35 % in water, TMS-EDTA) was purchased from Flurochem Ltd. (UK). *N*-[3-(Trimethoxysilyl)propyl]-*N,N,N*-trimethylammonium chloride (50 % in methanol,TMS-TMAC) was purchased from Alfa Aesar. Model organic solutes (rose bengal (RB), tris(bipyridine)ruthenium(II) chloride (Ru), orange II (OR−), naphthol green B (NG3−), reactive green 19 (RG6−) and β-Cyclodextrin (CD0)) and proteins (lysozyme (LZ) and β-lactoglobulin (LG)) were purchased from Sigma-Aldrich.

1.2. Synthesis of diblock copolymers PS-*b*-P4VP

PS-*b*-P4VP diblock copolymers were synthesized by sequential living anionic polymerization following a previously published procedure,[1] as shown in Scheme 1. The polymerization of styrene was initiated by *s*-BuLi in THF at −78 °C for approximately 2 h. Afterwards, the purified 4VP was added to the reactor via a syringe and polymerized onto the living polystyrene for approx. 14 h. The polymerization was terminated with degassed methanol/acetic acid (90/10 by volume, v/v). After removal of THF under reduced pressure and precipitation in water, the PS-*b*-P4VP diblock copolymer was obtained.

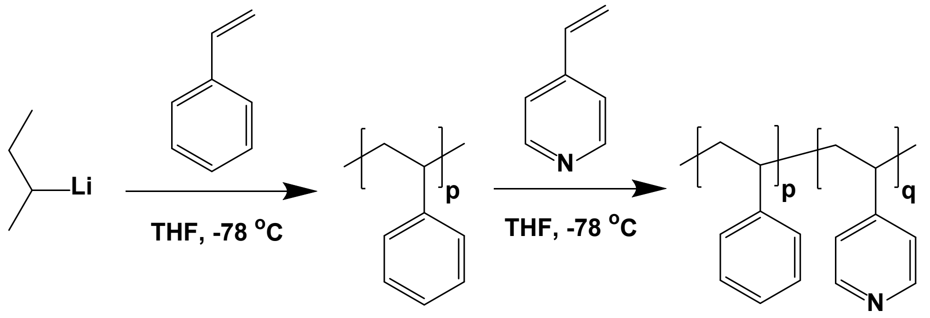

Scheme 1. Synthesis route of PS-*b*-P4VP diblock copolymers by living anionic polymerization.

1.3. SNIPS membrane fabrication procedure

Integral asymmetric isoporous membranes were prepared by the SNIPS technique. In general, the PS-*b*-P4VP diblock copolymers were dissolved in a solvent mixture of THF/*N,N*-dimethylformamide (DMF) (40:60 wt%). After stirring for 24 h, the casting solutions were directly cast on a polyester nonwoven support using a doctor blade with a gap height of 200 µm. The films were left for a certain time (5–20 s) under air before immersing them into a non-solvent bath (water bath). After immersing for several hours in the water bath, the membranes were dried at 60 °C in a vacuum oven.

1.4. AlO_x growth by sequential infiltration and atomic layer deposition

AlO_x was grown on the porous membranes using a Veeco Savannah S100 ALD system. Growth was proceeded with a 10 min stabilization step, where samples were held in the ALD chamber at 95 °C under a 20 sccm flow of nitrogen with a 0.3 Torr pressure environment. Trimethylaluminum (TMA) was used as the Al source precursor and water as the co-reactant. SIS was performed at semi-static mode with continuous nitrogen flow during the exposure step. An SIS cycle of AlO_x consisted of: 15 ms TMA pulse/300 s exposure (in semi-static mode)/350 s N_2 purge/15 ms water pulse/300 s exposure (in semi-static mode)/350 s N_2 purge. The pressure during the SIS process was measured with a Pirani gauge. From a comparison between a Pirani gauge and a capacitance gauge, we estimate the chamber pressure as ~50 Torr during TMA exposure and ~40 Torr during water exposure. These pressures represent a

combination of the precursor vapor pressure and nitrogen pressure and not the partial pressure of the TMA or water alone. An ALD cycle of AlO_x consisted of: 15 ms TMA pulse/10 s hold /10 s N_2 purge/15 ms water pulse/10 s hold/ 10 s N_2 purge. The nitrogen flow rate was set to 5 sccm during pulse and exposure steps of SIS and ALD and 20 sccm during the purge steps.

1.5. Post-functionalization of AlO_x modified membranes

Post-functionalization of AlO_x modified membranes was accomplished by silanization with different agents, *i.e.*, perfluorododecyltrichlorosilane (FDTS), *N*-[(3-trimethoxysilyl)propyl]ethylenediamine triacetic acid trisodium salt (35 % in water, TMS-EDTA) and *N*-[3-(trimethoxysilyl)propyl]-*N,N,N*-trimethylammonium chloride (50 % in methanol, TMS-TMAC).

FDTS treatment was performed by placing the membranes into FDTS vapour within a properly dried and evacuated desiccator at room temperature for 14-20 h. The resulting membranes were dried at 60 °C in a vacuum oven for 2 days. TMS-EDTA and TMS-TMAC treatments were carried out by immersing the membranes into a mixture of ethanol/H_2O (50:50-55:45 v/v) with a predetermined amount of TMS-EDTA and TMS-TMAC at room temperature under shaking of 120 rpm for 6 h. The resulting membranes were washed three times with ethanol/H_2O under shaking of 120 rpm to remove the unreacted alkoxysilane, and then dried at 60 °C in a vacuum oven for 2 days.

1.6. Characterization

The chemical composition of the block copolymers was determined by ^1H NMR, which was performed on a Bruker Advance 300 NMR spectrometer at 300 MHz using deuterated chloroform ($CDCl_3$) as a solvent. Molecular weights and dispersity indices of the polymers were determined by gel permeation chromatography (GPC). The measurements were performed at 50 °C in THF or dimethylacetamide (DMAc) with LiCl using 3 µm PSS SDV gel columns at a flow rate of 1.0 mL min^{-1} (VWR-Hitachi 2130 pump, Hitachi, Tokyo, Japan). A

Waters 2410 refractive-index detector (λ = 930 nm) with a polystyrene (PS) calibration was used. Fourier transform infrared spectroscopy (FTIR) was conducted to detect the post-functionalization reaction using a Bruker Alpha (diamond-ATR unit).

The membrane morphology was investigated by scanning electron microscopy (SEM) and transmission electron microscopy (TEM). SEM images were taken on a Merlin (ZEISS, Oberkochen, Germany) at a voltage of 1.3 kV or 3 kV. The samples for secondary electron images were coated with ca. 1 nm platinum, while the samples for backscattered electron (BSE) images were coated with about 8 nm carbon. Cross-sectional SEM samples were prepared by Ar-ion milling (Precisions Etching and Coating System PECS II, Gatan Inc., USA), followed by coating of a 4 nm thick layer of carbon on the obtained cross-sections with the same device. TEM images were taken on a Tecnai G^2 F20 transmission electron microscope (Thermo Fisher Scientific, USA) with an acceleration voltage of 120 kV in the bright-field mode. Cross-sectional TEM samples were produced by embedding the membrane samples in an epoxy resin (EPO-TEK®) followed by preparing ultrathin slices (~50 nm) using a Leica Ultra-microtome EM UCT system (Leica Microsystems, Wetzlar, Germany), with a diamond knife (Diatome Ltd., Switzerland). Average pore size values were determined using the software IMS (Imagic Bildverarbeitung AG, Switzerland) on the basis of the SEM results. Energy dispersive X-ray spectroscopy (EDX) measurements were performed on modified membranes by using the aforementioned SEM system, equipped with an EDX detector (X-Max 150 mm^2, Oxford Instruments, U.K.), at a working distance of 6 mm, a constant magnification of 5kx and an acceleration voltage of 5 kV.

Membrane surface zeta potential was determined using a SurPASS 3 electrokinetic analyzer (Anton Paar, Austria) with a background of 1 mM NaCl solution. The streaming channel gap height was adjusted at 100 µm. The pH values were adjusted using 50 mM HCl and 50 mM NaOH solutions in a range of 4.0-7.5. Each data point was measured 4 times. The zeta potential

(ζ) was calculated from the streaming potential (U_{str}) using the Helmholtz-Smoluchowski equation:

$$\zeta = \frac{dU_{str}}{d\Delta p}\frac{\eta}{\varepsilon\varepsilon_0}\frac{L}{A}\frac{1}{R} \quad (1)$$

where U_{str} is the streaming potential, Δp is the hydrodynamic pressure difference across the streaming channel, η is the viscosity of the electrolyte solution, ε is the permittivity of the electrolyte solution, ε_0 is the vacuum permittivity, L is the length of the streaming channel, A is the cross-section of the streaming channel, and R is the electrical resistance inside the streaming channel.

Hydrophilicity of the membranes was assessed by the dynamic water contact angle using a KRUESS Drop Shape Analysis System (DSA 100) or goniometer (Data Physics; OCA 15Pro). The contact angle over time was measured with 5 µL water droplet in a sessile drop manner. Each sample measurement was repeated at least 3 times.

Thermal stability of the membranes was estimated by thermal annealing of the membrane under 100-150 °C for 2 h in a vacuum oven. Afterwards, the water permeance and membrane structure of the heat-treated membranes were determined.

1.7. Membrane performance test

1.7.1. *Water permeance*

Water permeance measurements were performed in dead-end mode using a home-made automatic testing device at a transmembrane pressure (Δp) of 1 bar at room temperature. The volume change ΔV was measured gravimetrically for time slots Δt of 1-3 min for 2 h. The effective membrane area A was 1.77 cm^2. These studies were carried out by employing ultrapure water with an electrical conductivity of ≈ 0.055 µS cm^{-1} and a density of 0.998 g cm^{-3} at room temperature (20-22 °C). A minimum of 3 samples were measured. The water permeance (J_w) was calculated by normalizing the flux by the transmembrane pressure.

The water permeance was calculated as follows:

$$J_w = \frac{\Delta V}{A \Delta t \Delta p} \quad (2)$$

1.7.2. ***Separation performance***

The separation performance was evaluated by following a similar procedure used in previous studies.[2] Briefly, retention measurements were carried out using a stirred test cell (EMD Millipore™ XFUF07601, effective membrane area 1.13 cm^2) at a transmembrane pressure of 1 bar at room temperature. Prior to the retention test, the ultrapure water initially passed through the membranes for 1 h to stabilize the porous structure of membranes. Aqueous solutions of model organic small molecules were used at a concentration of 0.1 mM while aqueous solutions of proteins were at a concentration of 0.5 g L^{-1}. To minimize the effects of concentration polarization, the feed solution was stirred at 500 rpm during measurements. The permeance and retention were determined when the membrane reached a steady state. Measurements were repeated on a minimum of 3 samples.

The concentration of the solute except β-cyclodextrin (CD0) in the feed C_f (mg L^{-1}), permeate C_p (mg L^{-1}) and retentate C_r (mg L^{-1}) was determined by a UV-vis spectrophotometer (GENESYS 10S, Thermo Scientific), while the concentration of CD0 was detected by GPC. The retention (*R, %*) of the solute was calculated using Equation 3:

$$R = \left(1 - \frac{C_p}{(C_f + C_r)/2}\right) \times 100 \quad (3)$$

Mixed-solute separation measurements were performed using the same procedure as mentioned above. The molar composition between two solutes is 1:1, but the total solute concentration in the feed was kept at 0.1 mM.

To quantify the figure of merit for molecular separation, we calculated the selectivity ψ, defined as the ratio of permeation of two solutes using Equation 4:

$$\psi = \frac{1-R_1}{1-R_2} \tag{4}$$

where R_1 and R_2 were observed retention values of two different solutes.

2. Results and Discussion

2.1. Tuning isoporous membrane pore size by AlO_x growth

Table S1. Molecular characterization of the polymers used for the membrane preparation

	PS [wt %][a)]	P4VP [wt %][a)]	M_n [kg mol^{-1}][b)]	M_w [kg mol^{-1}][b)]	Dispersity[c)]
PS-*b*-P4VP for 38 nm series	80	20	182	187	1.03
PS-*b*-P4VP for 55 nm series	76	24	261	274	1.05

[a)]Composition of polymers calculated from ^{1}H NMR spectra; [b)]Molecular weight calculated based on ^{1}H NMR spectra and GPC; [c)]Dispersity determined by GPC.

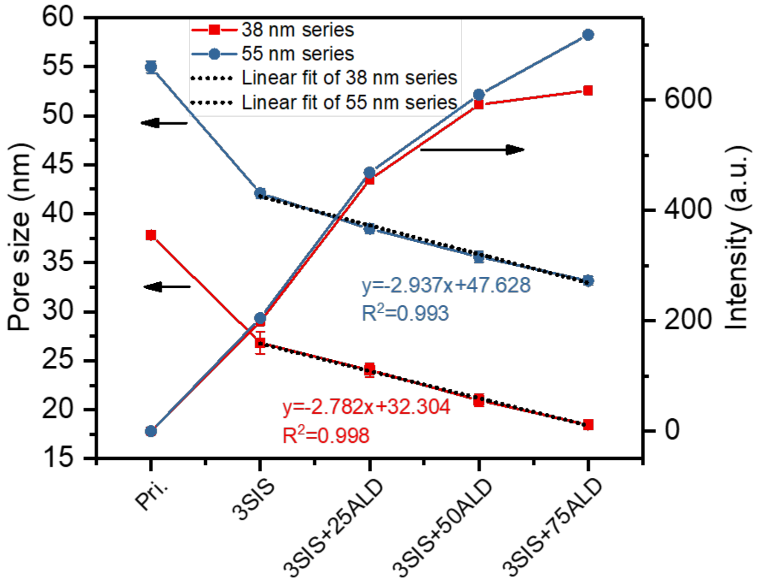

Figure S1. Effect of AlO_x growth on the pore size of two series of membranes (38 nm and 55 nm series) measured from SEM images (left Y-axis). AlO_x growth with SIS and ALD cycles determined by EDX using the absolute intensity of the Al peak at 1.49 eV (right Y-axis).

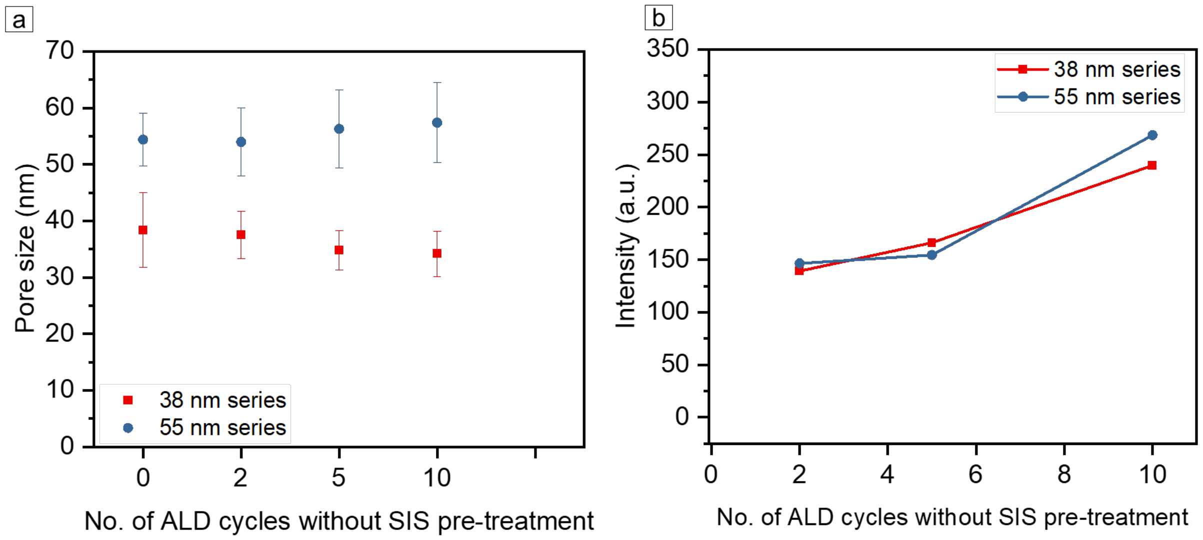

Figure S2. (a) Effect of AlO_x ALD growth without AlO_x SIS pre-treatment on the membrane pore size, calculated based on SEM images. (b) The amount of Al was determined by EDX using the absolute intensity of the Al peak at1.49 eV as a function of no. of ALD cycles without AlO_x SIS pre-treatment.

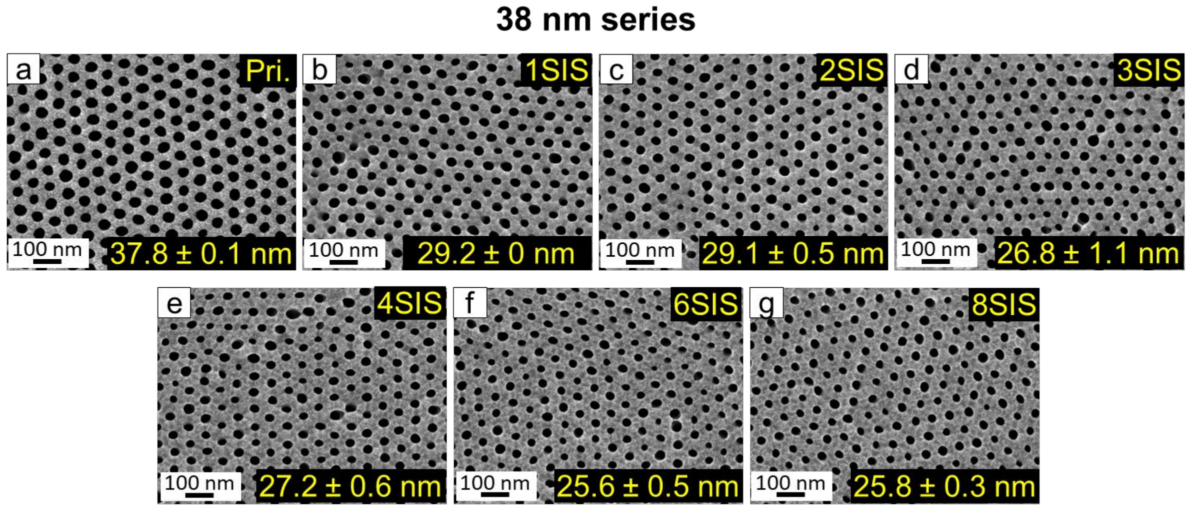

Figure S3. Effect of initial AlO_x SIS growth on the membrane pore size of the 38 nm series.

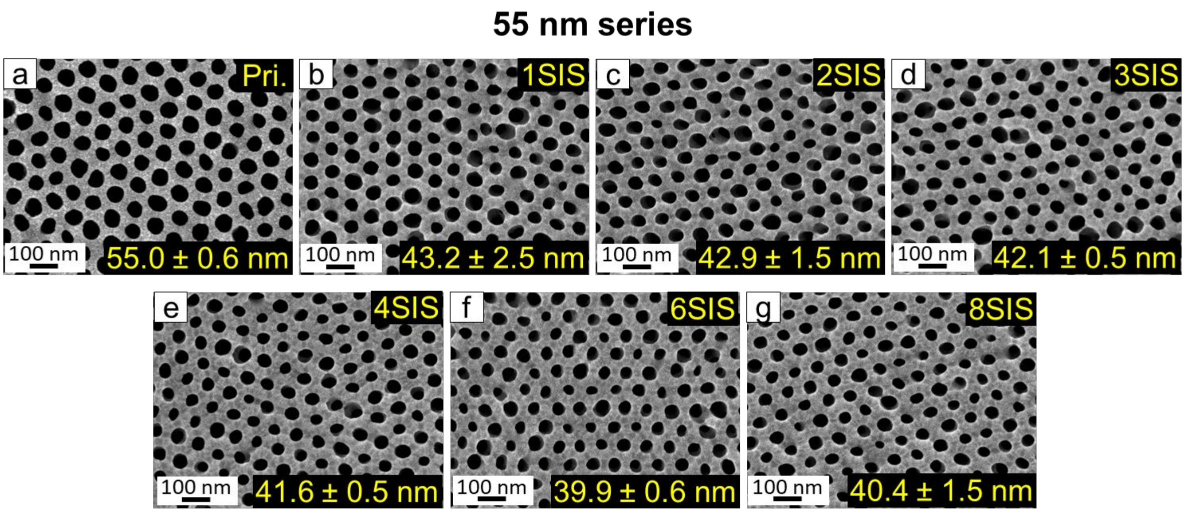

Figure S4. Effect of initial AlO_x SIS growth on the membrane pore size of the 55 nm series.

We investigated the effect of initial AlO_x SIS growth on the membrane pore size by varying the number of SIS cycles (Figure S3 and Figure S4). For both 38 nm series and 55 nm series, the pore diameter exhibits a significant reduction (around 9 - 12 nm) with merely 1 SIS cycle (Figure S3a, b and Figure S4a, b). Additional SIS cycles reduced the pore size at ~0.5 nm/cycle (38 nm series) and ~0.4 nm/cycle (55 nm series). Overall, the P4VP pore-forming block reached significant swelling/infiltration after 3-6 SIS cycles. Therefore, 3 SIS cycles was selected as the pre-treatment prior to ALD cycles.

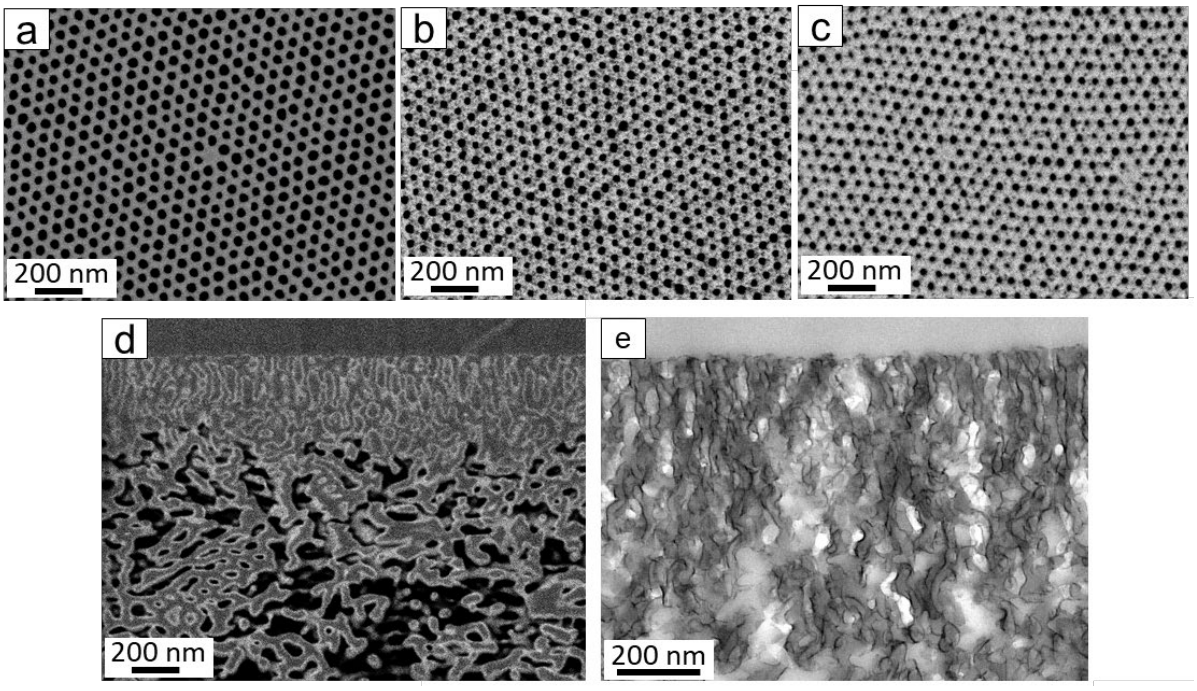

Figure S5. Backscattered electron (BSE) images of the (a-c) top surface and (d) cross-section: (a) Pristine 38 nm membrane (Pri.38), (b, d) 38 nm membrane modified with 3SIS+25ALD (38_3SIS+25ALD), (c) 38 nm membrane modified with 3SIS+75ALD (38_3SIS+75ALD). (e) Cross-sectional TEM image of 38_3SIS+25ALD.

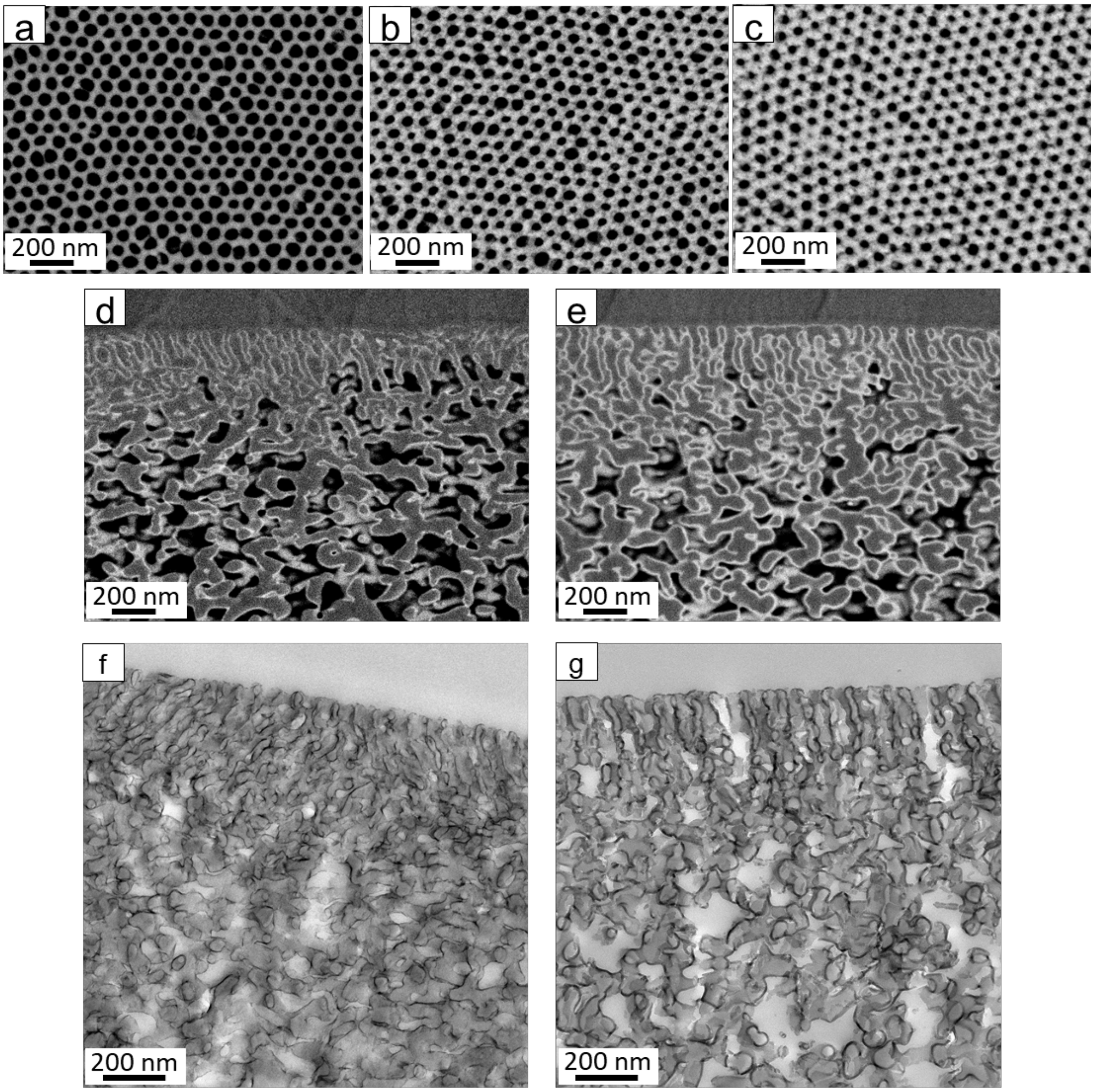

Figure S6. Backscattered electron (BSE) images of the (a-c) top surface and (d, e) cross-section of membranes: (a) Pristine 55 nm membrane (Pri.55), (b, d) 55 nm membrane modified with 3SIS+25ALD (55_3SIS+25ALD), and (c, e) 55 nm membrane modified with 3SIS+75ALD (55_3SIS+75ALD). TEM images of the membrane cross-section: (f) 55_3SIS+25ALD and (g) 55_3SIS+75ALD.

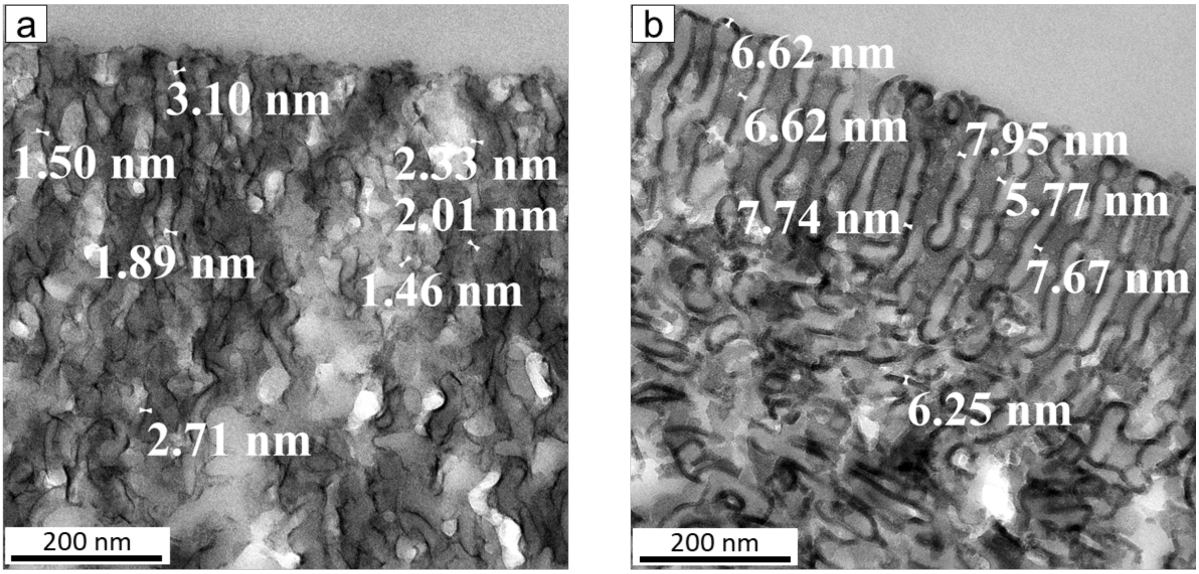

Figure S7. Thickness of the AlO_x growth layer from the top surface down to approx. 700 nm depth along the cross-section of the membrane, measured in TEM images: (a) 38_3SIS+25ALD, (b) 38_3SIS+75ALD.

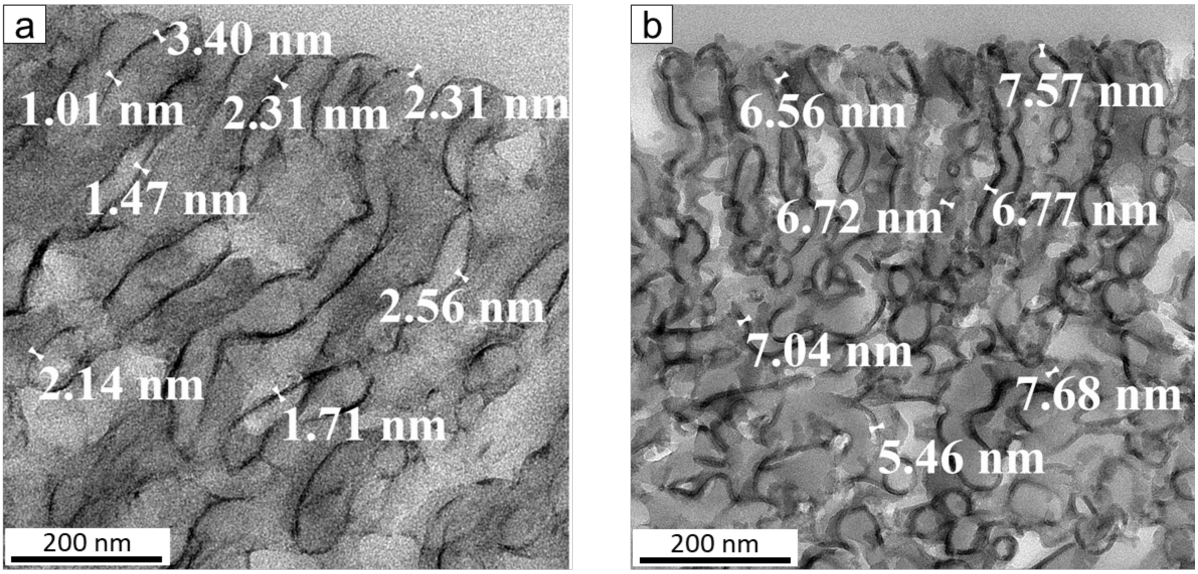

Figure S8. Thickness of the AlO_x growth layer from the top surface down to approx. 700 nm depth along the cross-section of the membrane, measured in TEM images: (a) 55_3SIS+25ALD, (b) 55_3SIS+75ALD.

Table S2. Average thickness of the AlO_x growth layer from the top surface down to approx. 700 nm depth along the cross-section of membrane.

	3SIS+25ALD	3SIS+75ALD
38 nm series	2.08 ± 0.47 nm	7.03 ± 0.59 nm
55 nm series	2.12 ± 0.52 nm	7.18 ± 0.80 nm

Each average value was calculated based on 3-4 TEM images, and each image had 7-8 measured values.

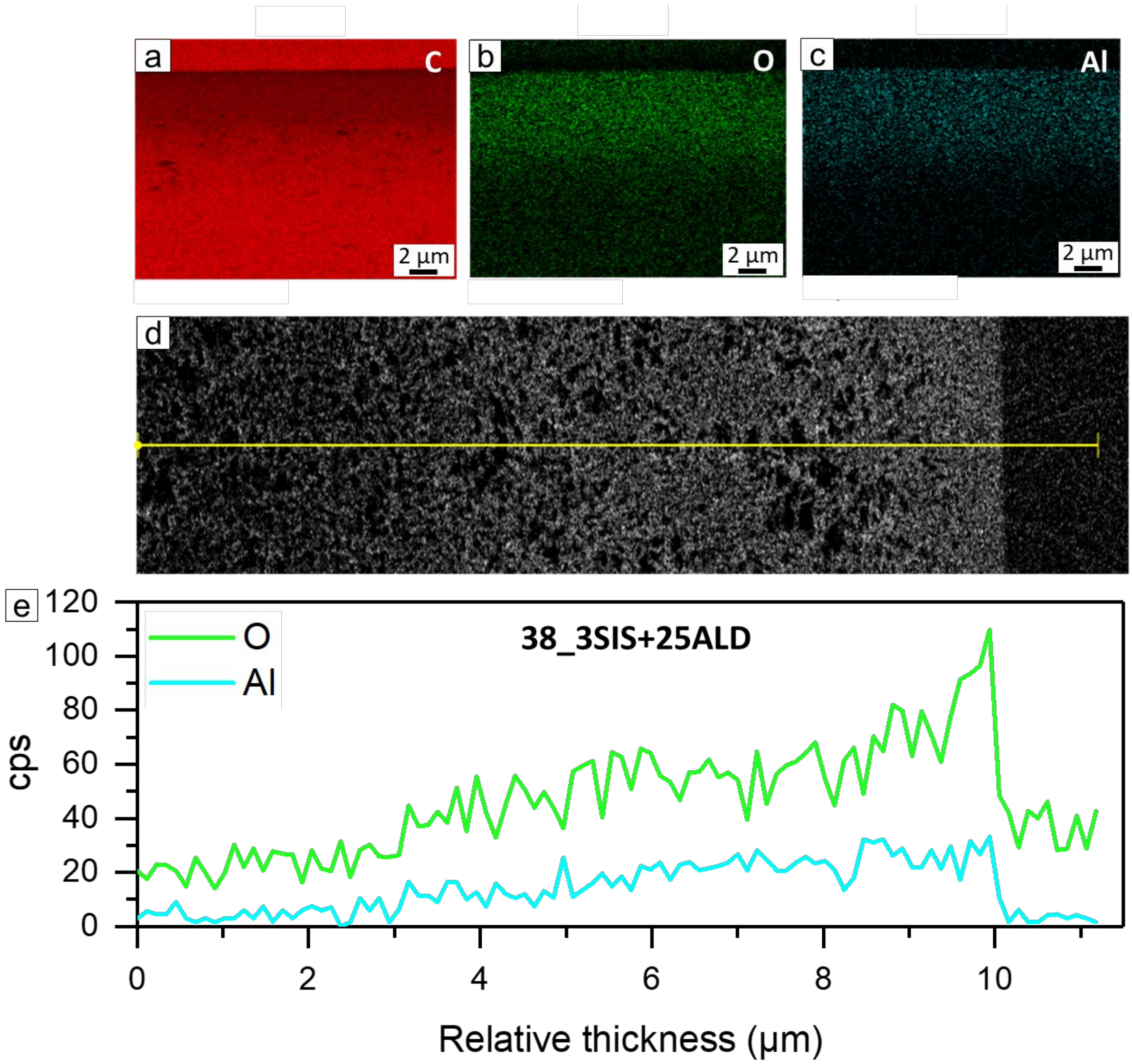

Figure S9. (a-c) The elemental distribution of carbon, oxygen and aluminum along the cross-section of 38_3SIS+25ALD. (d, e) A reconstructed elemental line scan of 38_3SIS+25ALD.

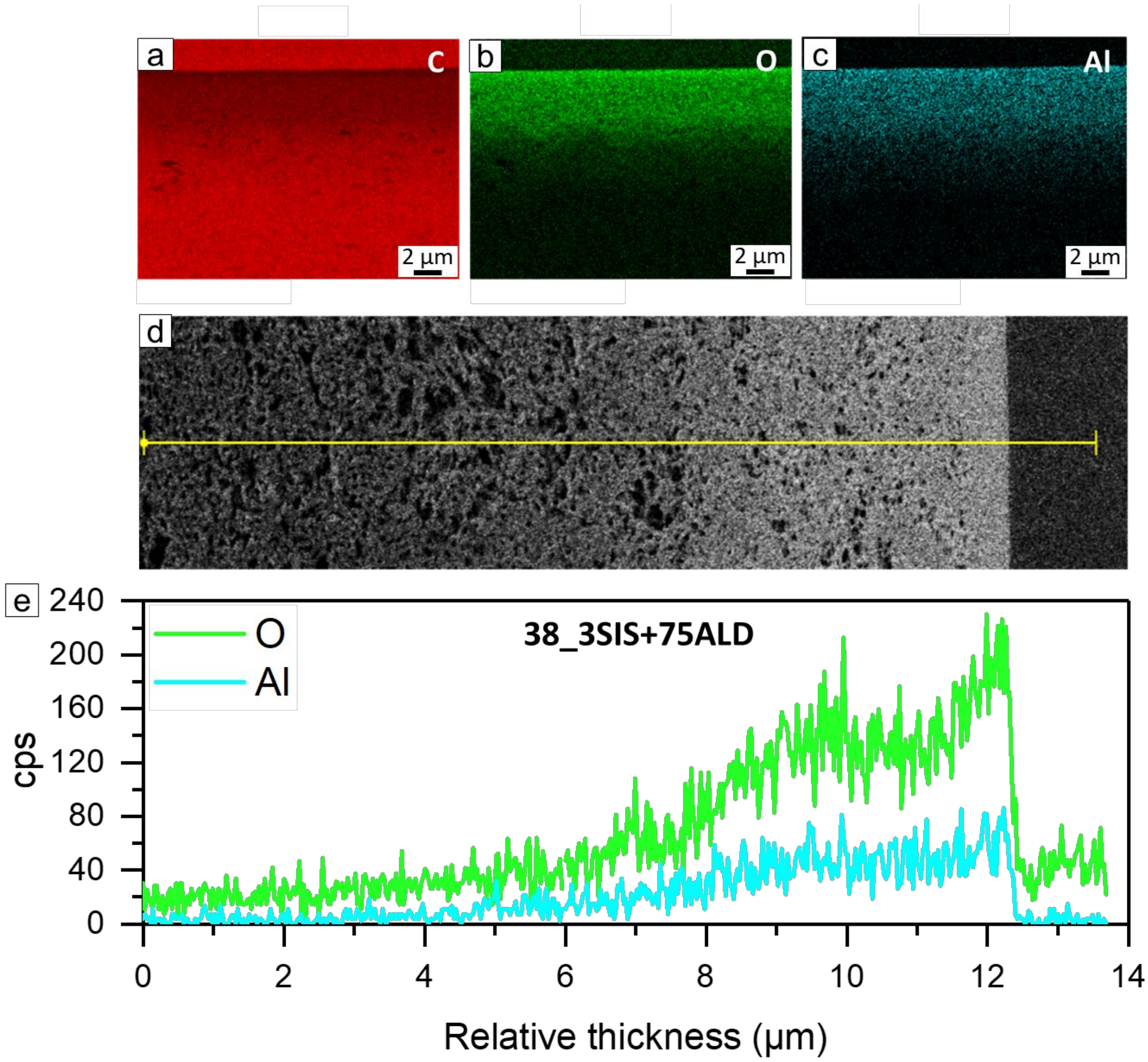

Figure S10. (a-c) The elemental distribution of carbon, oxygen and aluminum along the cross-section of 38_3SIS+75ALD. (d, e) A reconstructed elemental line scan of 38_3SIS+75ALD.

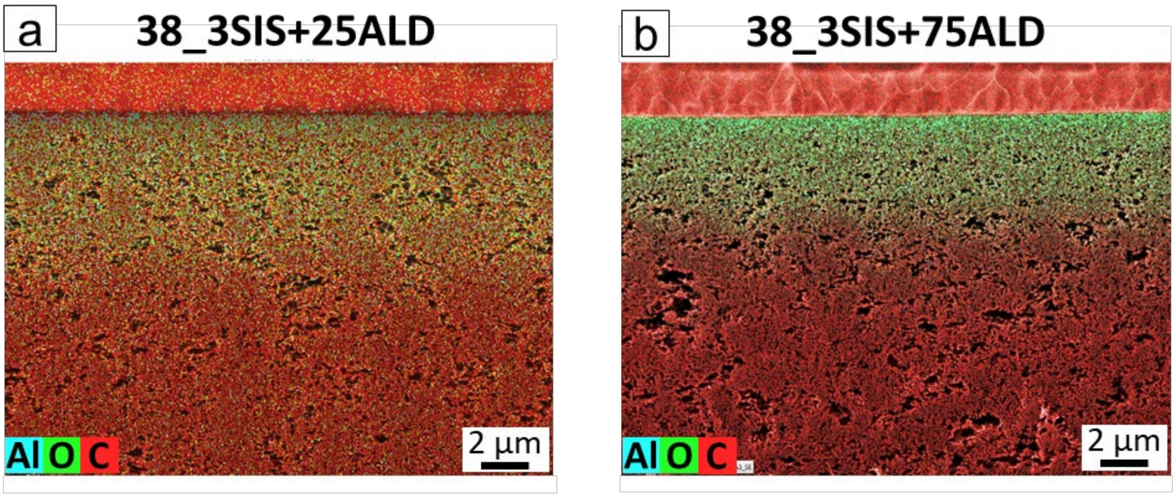

Figure S11. (a, b) The elemental distribution of carbon, oxygen and aluminum along the cross-section of 38_3SIS+25ALD and 38_3SIS+75ALD.

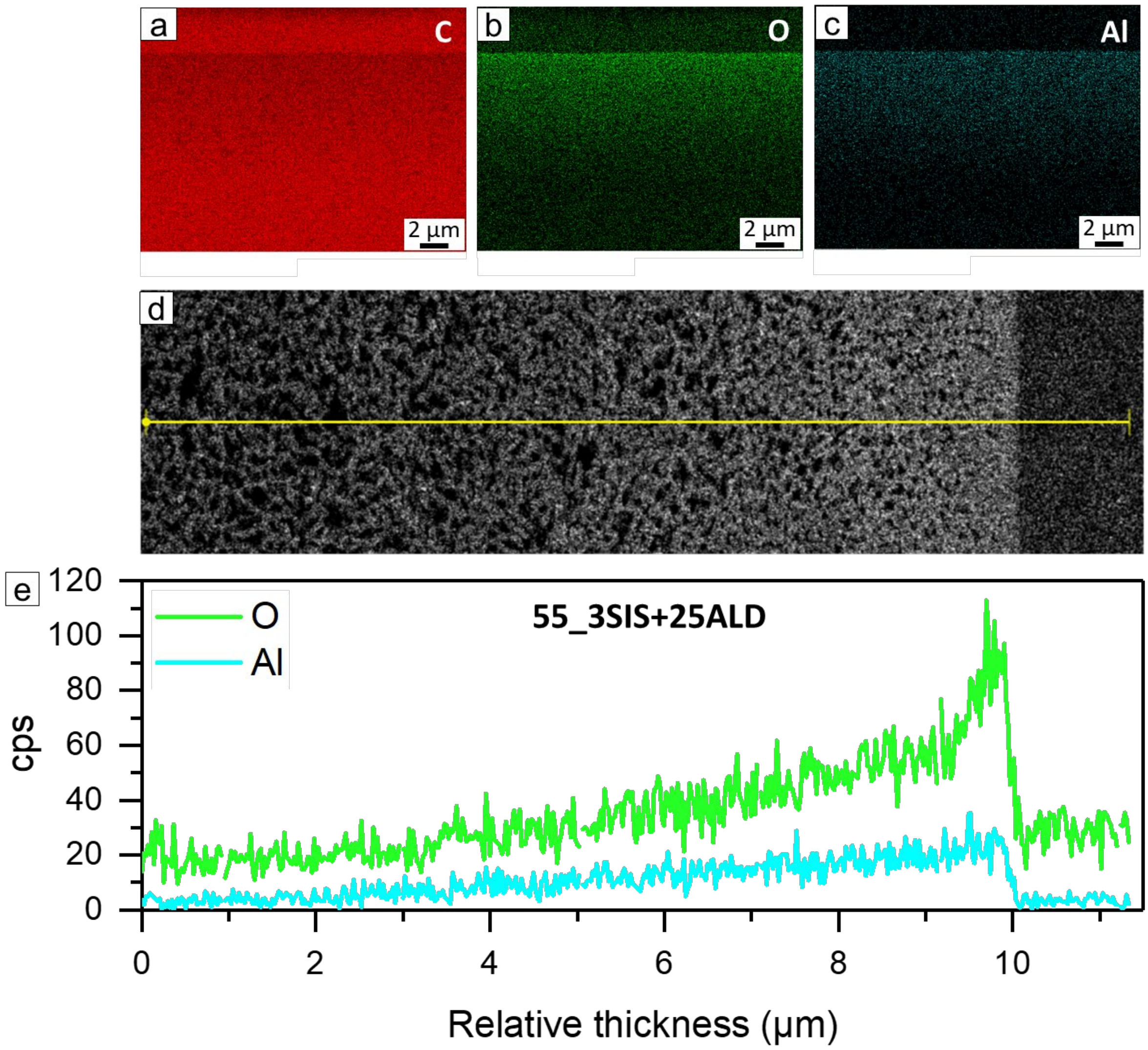

Figure S12. (a-c) The elemental distribution of carbon, oxygen and aluminum along the cross-section of 55_3SIS+25ALD. (d, e) A reconstructed elemental line scan of 55_3SIS+25ALD.

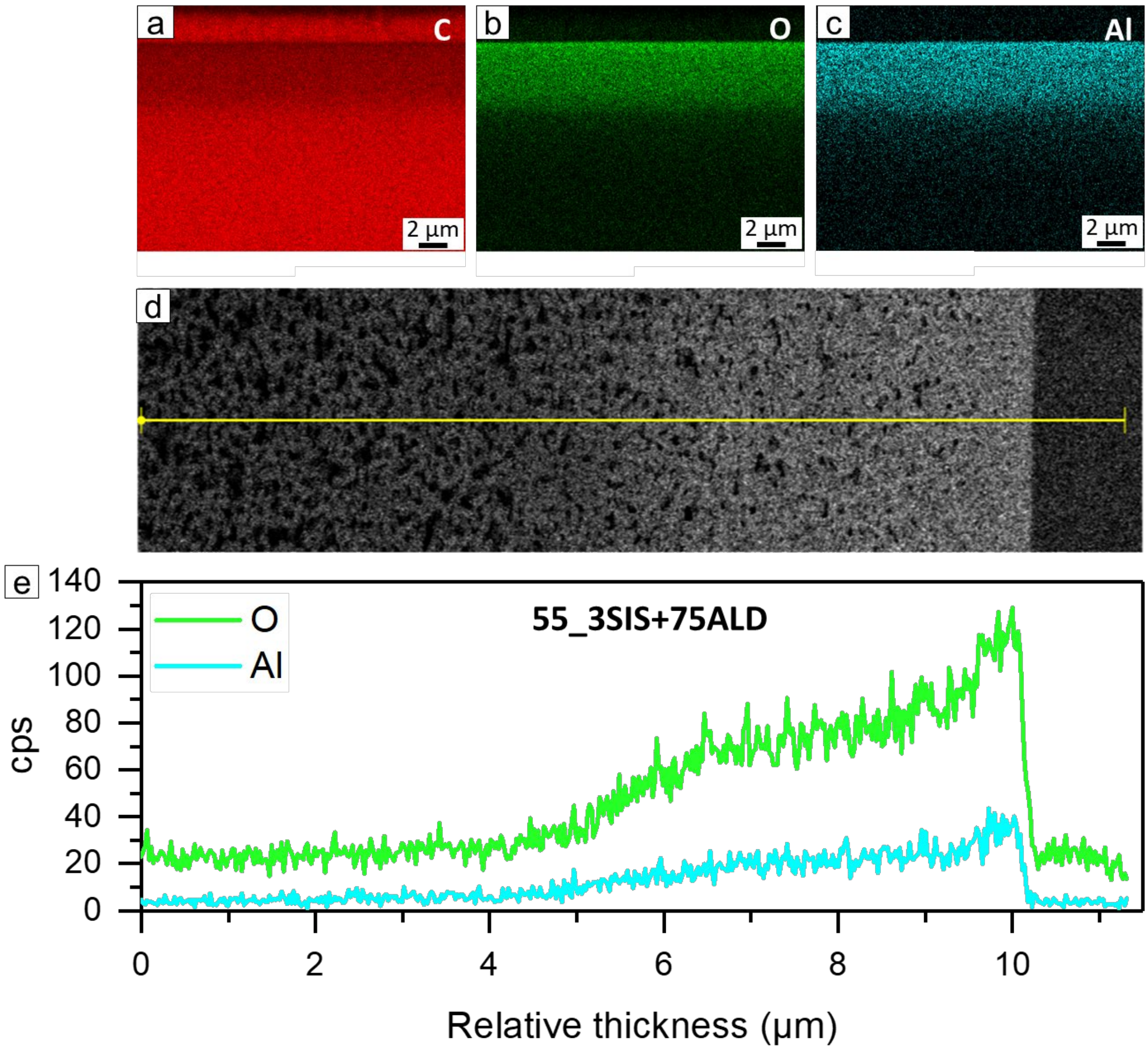

Figure S13. (a-c) The elemental distribution of carbon, oxygen and aluminum along the cross-section of 55_3SIS+75ALD. (d, e) A reconstructed elemental line scan of 55_3SIS+75ALD.

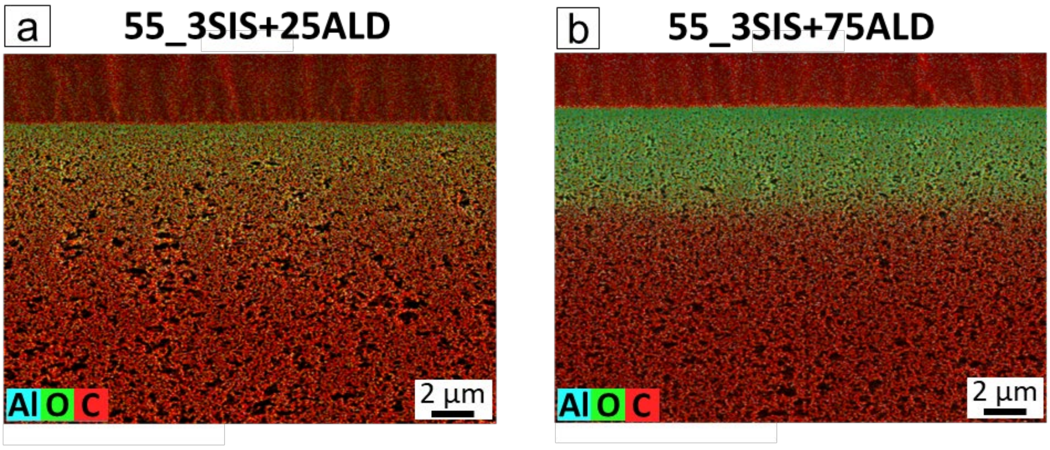

Figure S14. The elemental distribution of carbon, oxygen and aluminum along with the cross-section of 55_3SIS+25ALD and 55_3SIS+75ALD.

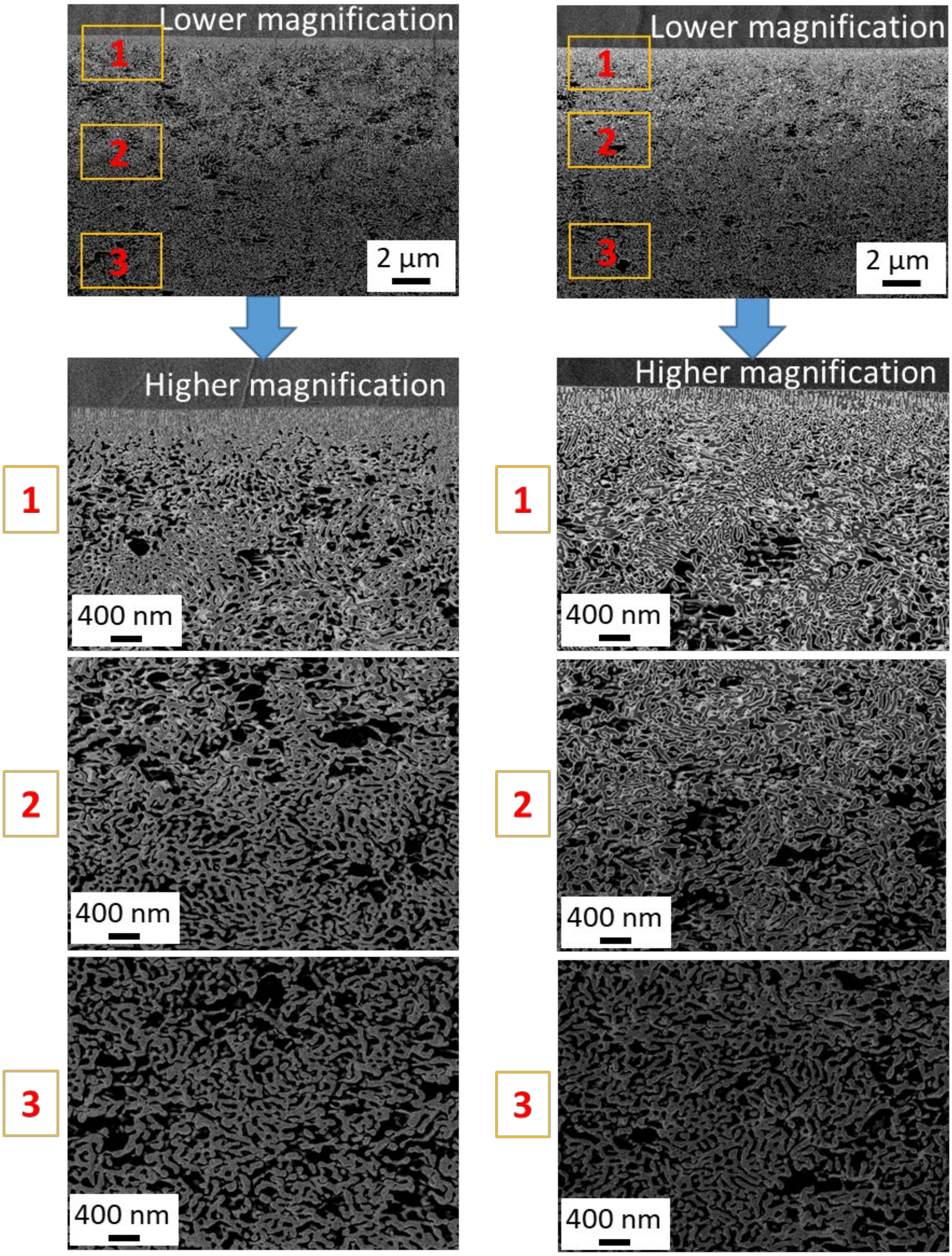

Figure S15. BSE images of the cross-section of membranes measured with different magnifications in different locations: Left column: 38_3SIS+25ALD. Right column: 38_3SIS+75ALD.

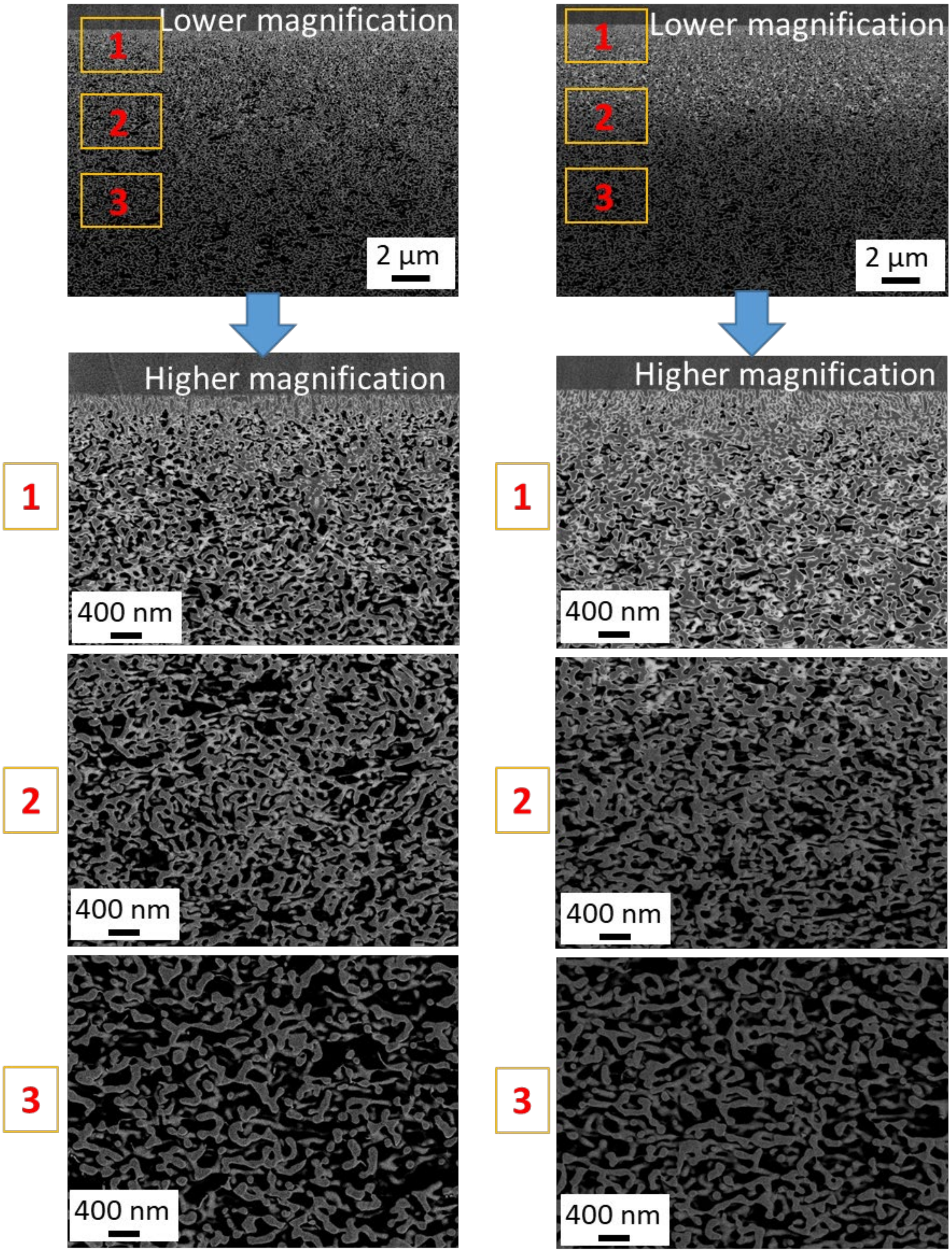

Figure S16. BSE images of the cross-section of membranes measured with different magnifications in different locations: Left column: 55_3SIS+25ALD. Right column: 55_3SIS+75ALD.

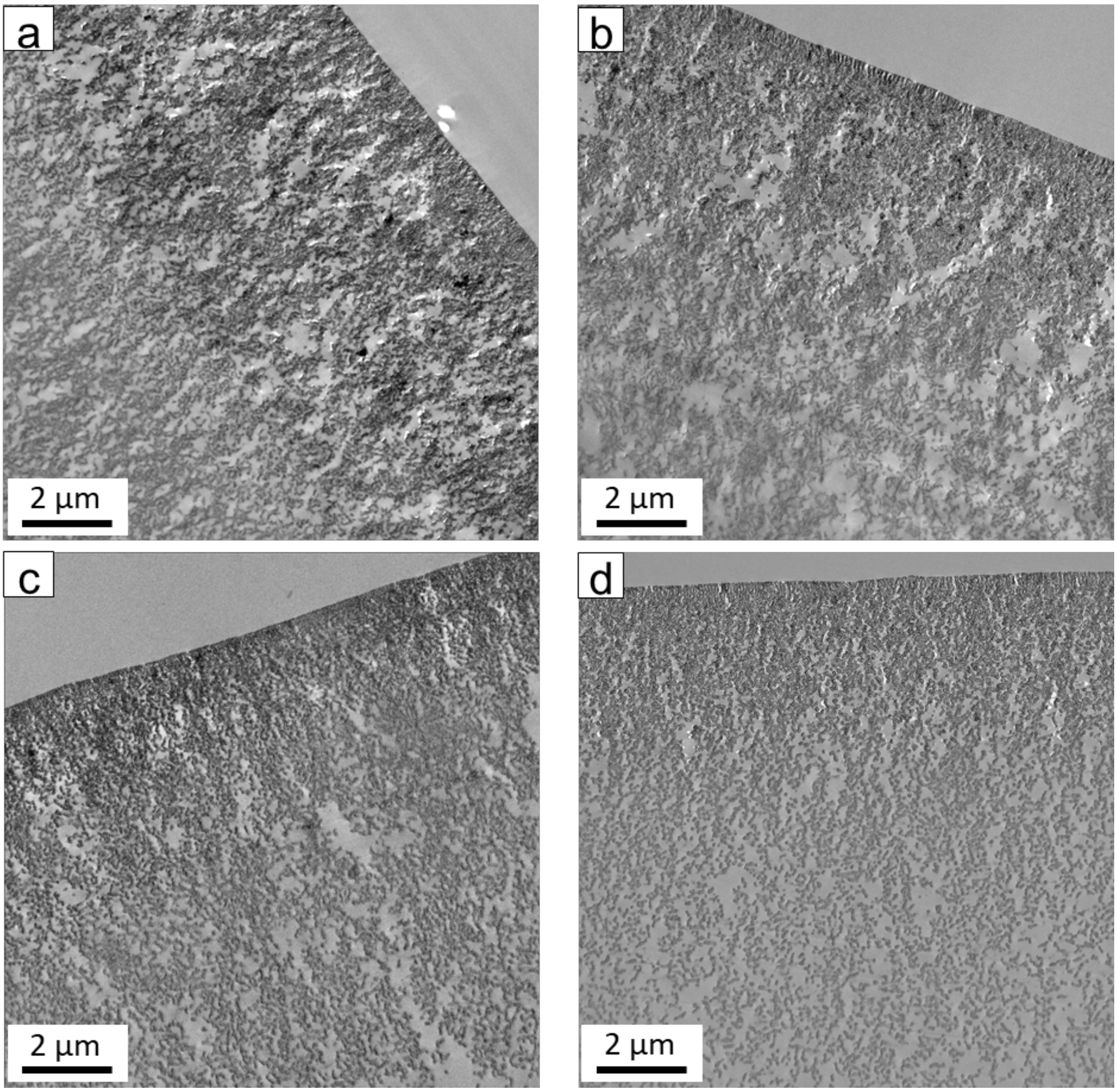

Figure S17. TEM images of the cross-section of the membranes with low magnification: (a) 38_3SIS+25ALD, (b) 38_3SIS+75ALD, (c) 55_3SIS+25ALD, (d) 55_3SIS+75ALD.

In order to reconfirm the distribution and penetration depth of the AlO_x layer along the cross-section of the membranes, Figure S15, Figure S16 and Figure S17 display the cross-sectional BSE and TEM images with low magnification.

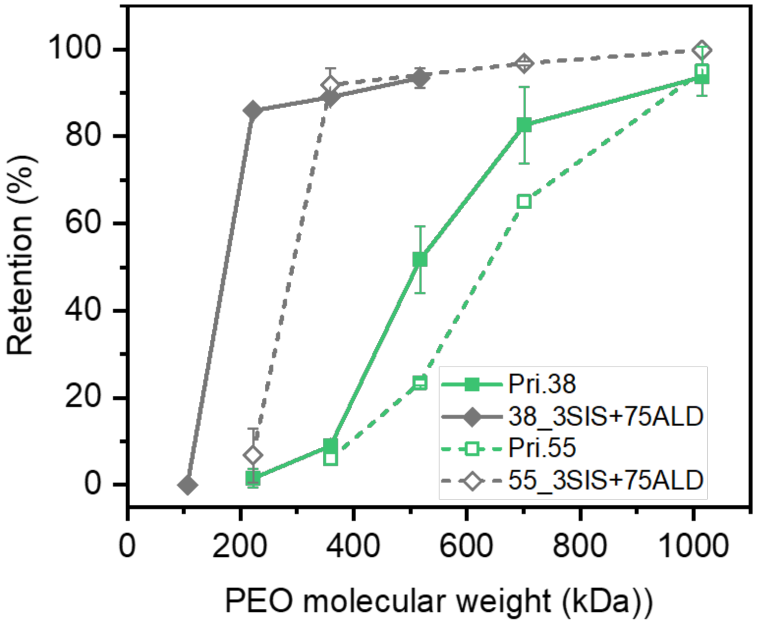

Figure S18. Retention of poly(ethylene oxide) (PEO) of the pristine membranes Pri.38 and Pri.55, and the 3SIS+75ALD modified membranes 38_3SIS+75ALD and 55_3SIS+75ALD.

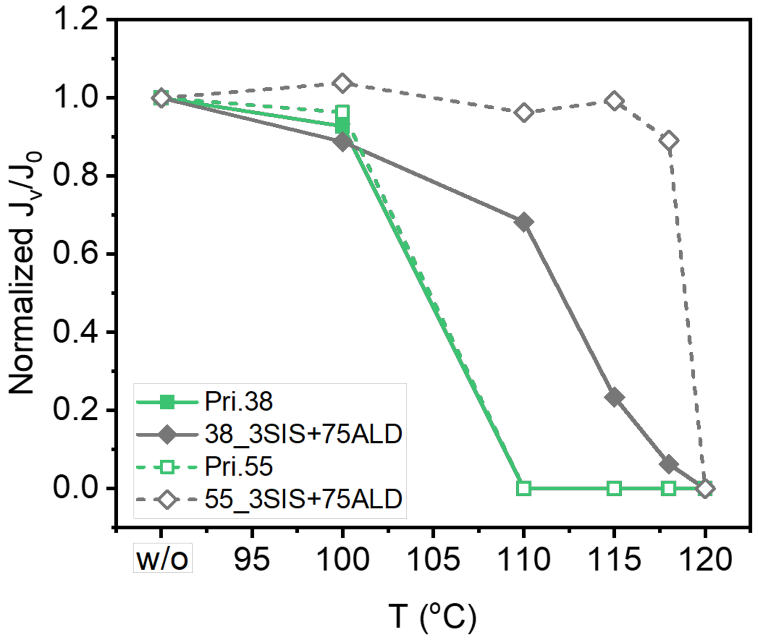

Figure S19. Normalized water permeance of Pri.38, 38_3SIS+75ALD, Pri.55, 55_3SIS+75ALD after the thermal annealing at temperatures of 100 °C, 110 °C, 115 °C, 118 °C and 120 °C for 2 h.

To investigate the thermal stability of the hybrid organic-inorganic isoporous membranes, the pristine PS-*b*-P4VP and 3SIS+75ALD treated membranes (*i.e.* Pri.38, 38_3SIS+75ALD, Pri.55, 55_3SIS+75ALD) were treated with different temperatures, *i.e.*, 100 °C, 110 °C, 115 °C, 118 °C, 120 °C, 130 °C, 140 °C and 150 °C. Figure S19-Figure S25 show the normalized water permeance and porous structure of these thermally annealed membranes. Both Pri.38 and Pri.55 retained more than 90 % permenace after 100 °C treatment, dropping to no permeance after ⩾ 110 °C treatments, since the corresponding cross-sectional structure was sintered to form a completely dense layer at ⩾ 110 °C (Figure S19, Figure S22 and Figure S24). In the case of the 3SIS+75ALD treated membranes, all thermally annealed membranes maintained the original isoporous surface, even at 150 °C (Figure S21). However, the membrane cross-section with 120 °C treatment started to get denser and exhibit sintering spots, while the cross-section with ⩾ 130 °C treatments had a completely dense layer underneath the AlO_x infiltrated area (Figure S23 and Figure S25). Consequently, after ⩾ 120 °C treatments, no water permeance was observed. Remarkably, 55_3SIS+75ALD preserved > 90 % permeance up to 118 °C. However, 38_3SIS+75ALD exhibited a gradual decrease with rising temperature (Figure S19). The slight sintering at 110 °C or 115 °C had an obvious impact on the water transport through the nanochannels of 38_3SIS+75ALD, presumably because the original cross-sectional structure of 38_3SIS+75ALD is denser than that of 55_3SIS+75ALD. From the above SEM, TEM and EDX investigations (Figure S6-Figure S17), AlO_x was not only grown on the selective layer of membrane, but penetrated the cross-section for 4-5 μm, which can stabilize the kinetically trapped porous structure under thermal annealing. Moreover, AlO_x growth was within the P4VP domain, not just on the surface, via initial SIS treatment, which made the porous structure tougher at high temperatures. Overall, the combined SIS and ALD provide a promising and facile top-down approach to fabricate thermally stable hybrid organic-inorganic isoporous membranes.

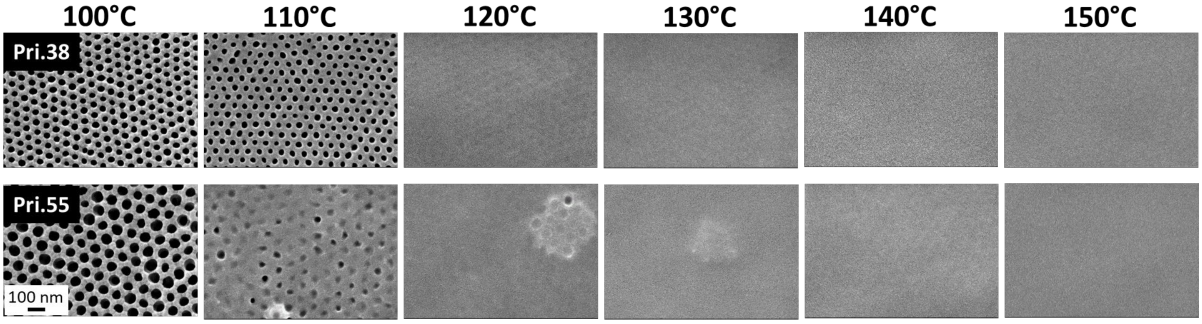

Figure S20. SEM images of the top surface of Pri.38, Pri.55 after thermal annealing at different temperatures for 2 h. All the images have the same scale bar.

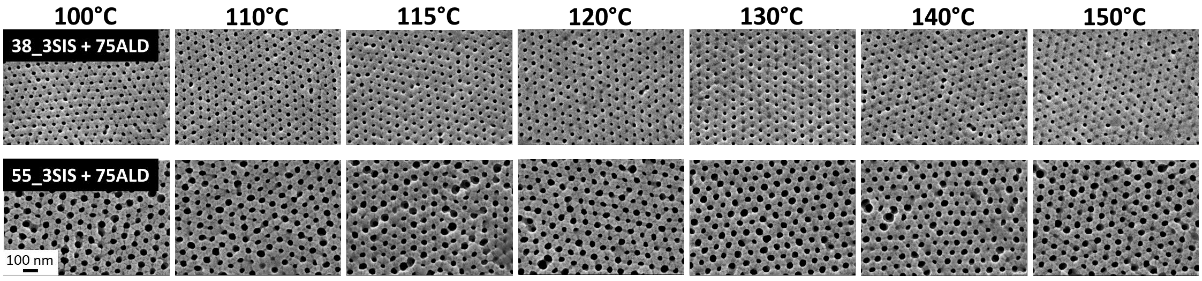

Figure S21. SEM images of the top surface of 38_3SIS+75ALD, 55_3SIS+75ALD after thermal annealing at different temperatures for 2 h. All the images have the same scale bar.

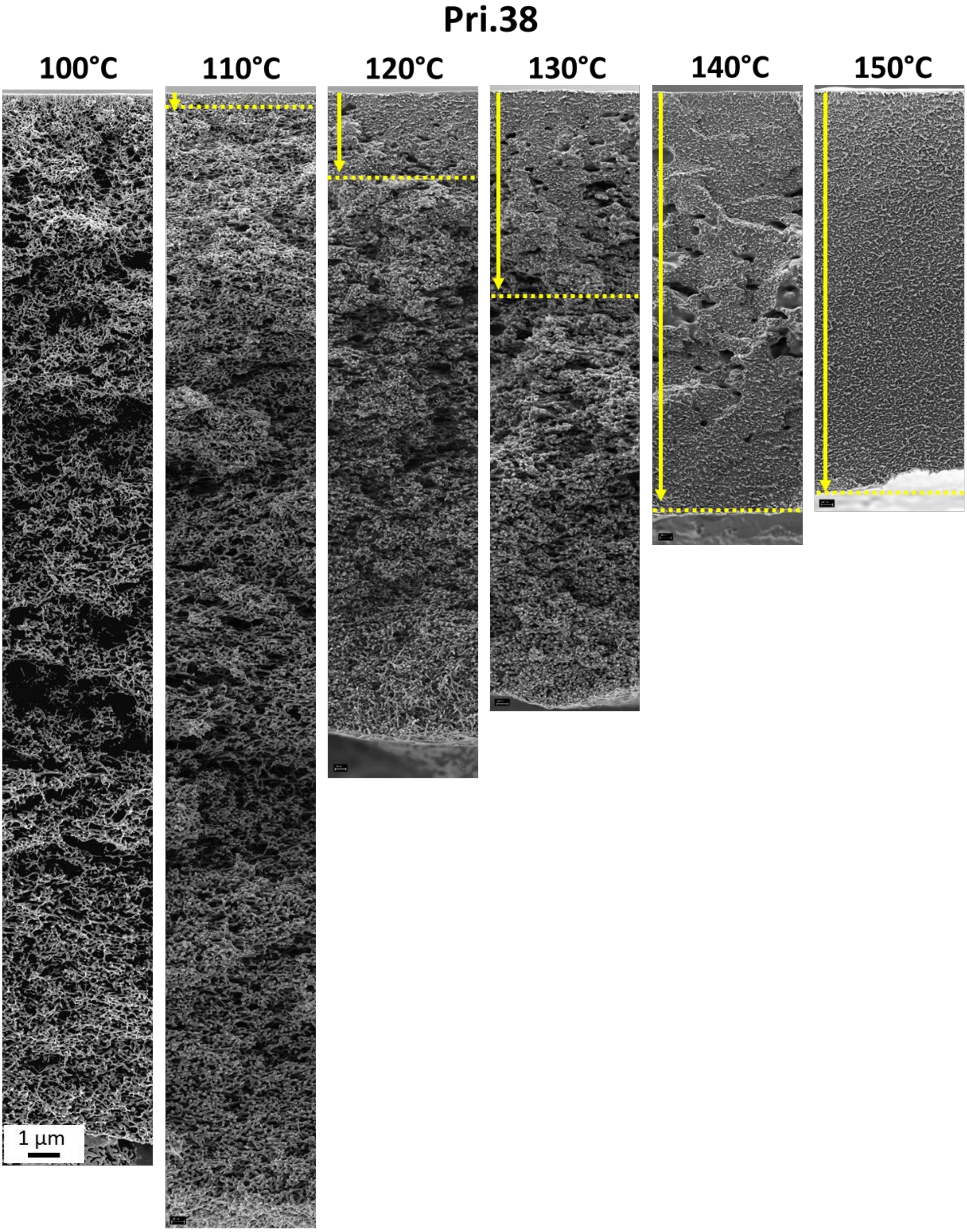

Figure S22. SEM images of the cross-section of Pri.38 after thermal annealing at different temperatures for 2 h. The area marked by the yellow arrow above the dashed line comprises a sintered dense structure. All the images have the same scale bar.

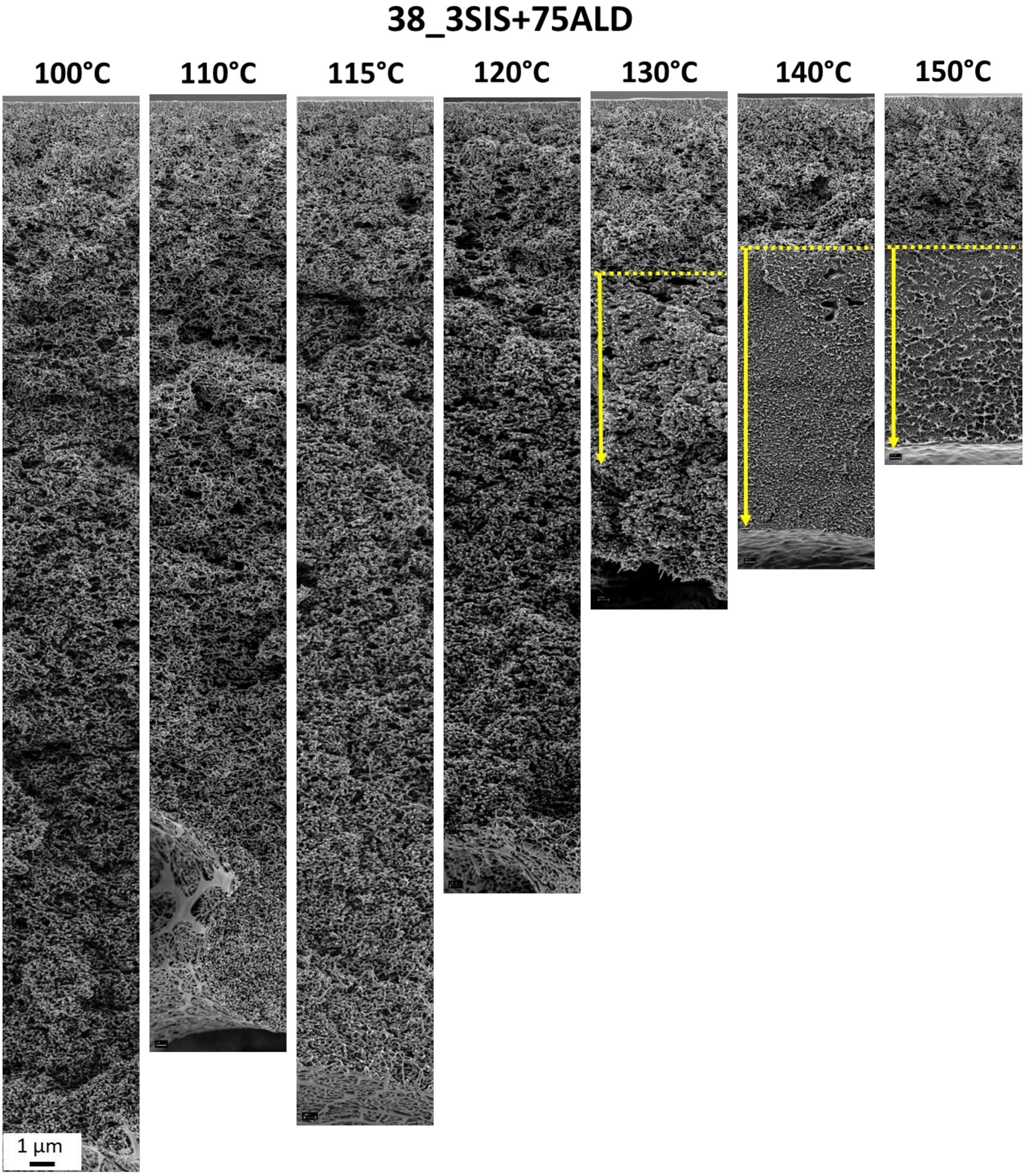

Figure S23. SEM images of the cross-section of 38_3SIS+75ALD after thermal annealing at different temperatures for 2 h. The area marked by the yellow arrow below the dashed line comprises a sintered dense structure. All the images have the same scale bar.

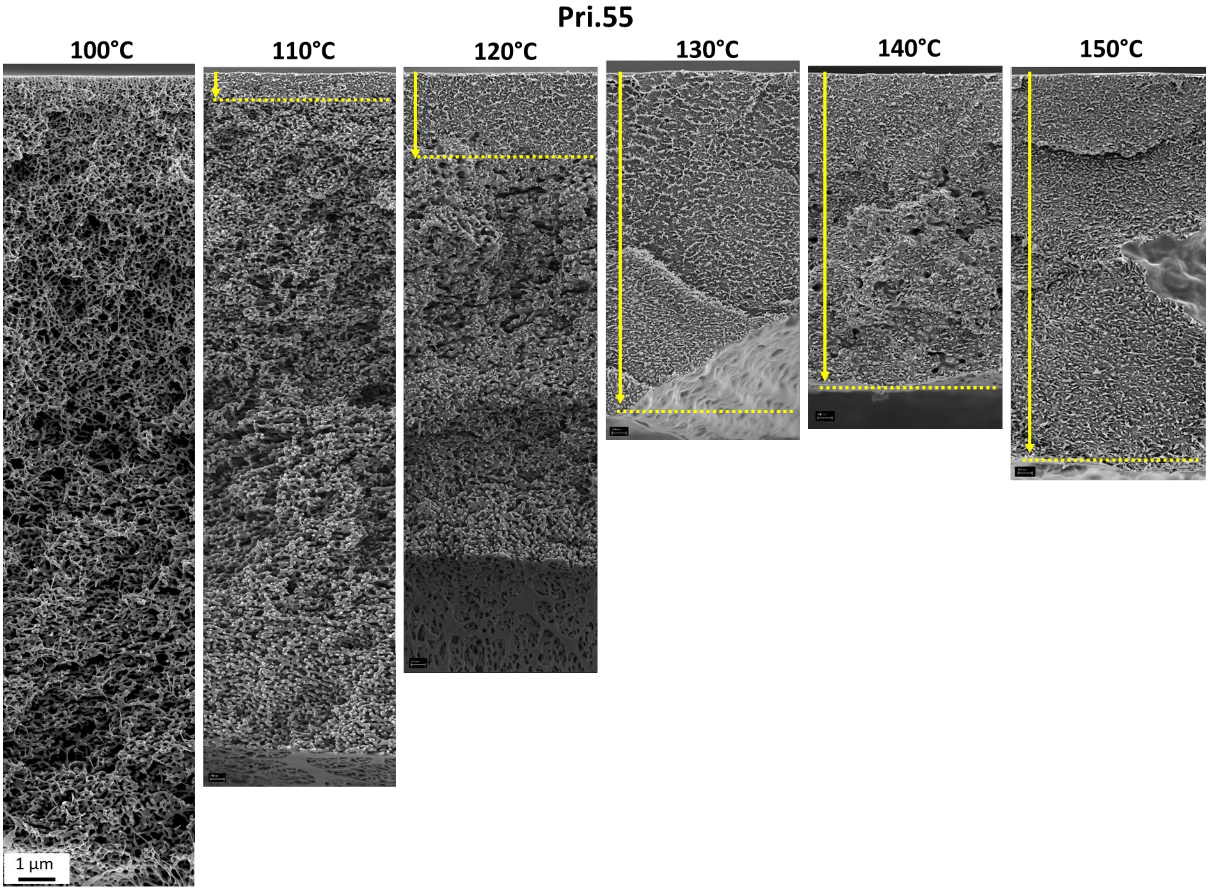

Figure S24. SEM images of the cross-section of Pri.55 after thermal annealing at different temperatures for 2 h. The area marked by the yellow arrow above the dashed line comprises a sintered dense structure. All the images have the same scale bar.

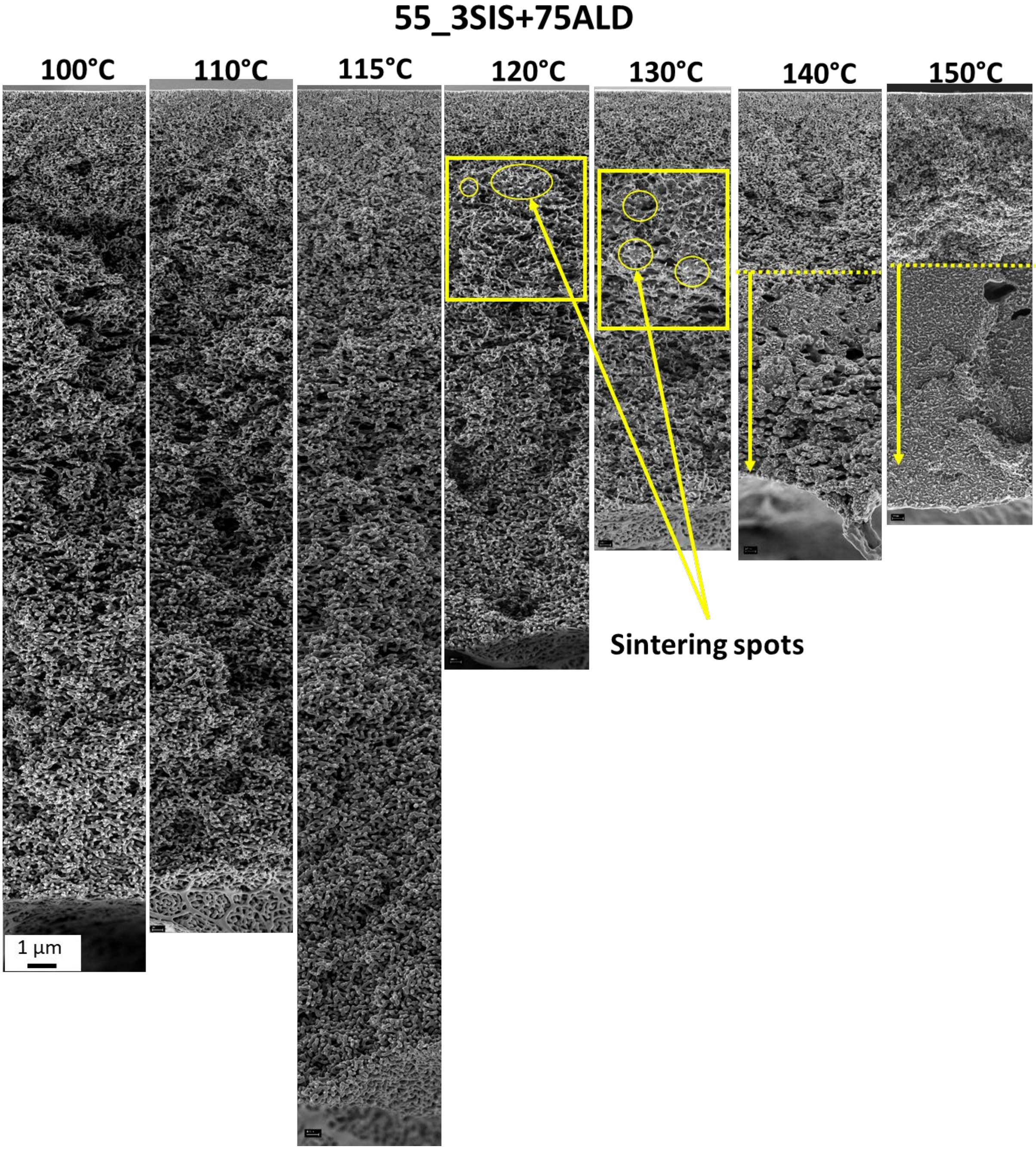

Figure S25. SEM images of the cross-section of 55_3SIS+75ALD after thermal annealing at different temperatures for 2 h. From 120 °C to 130 °C, the microstructure starts to develop sintering spots in the marked areas, which block the penetration of water. For 140 and 150 °C, the area marked by the yellow arrow below the dashed line comprises a sintered dense structure. All the images have the same scale bar.

2.2. Post-functionalization of hybrid organic-inorganic isoporous membranes

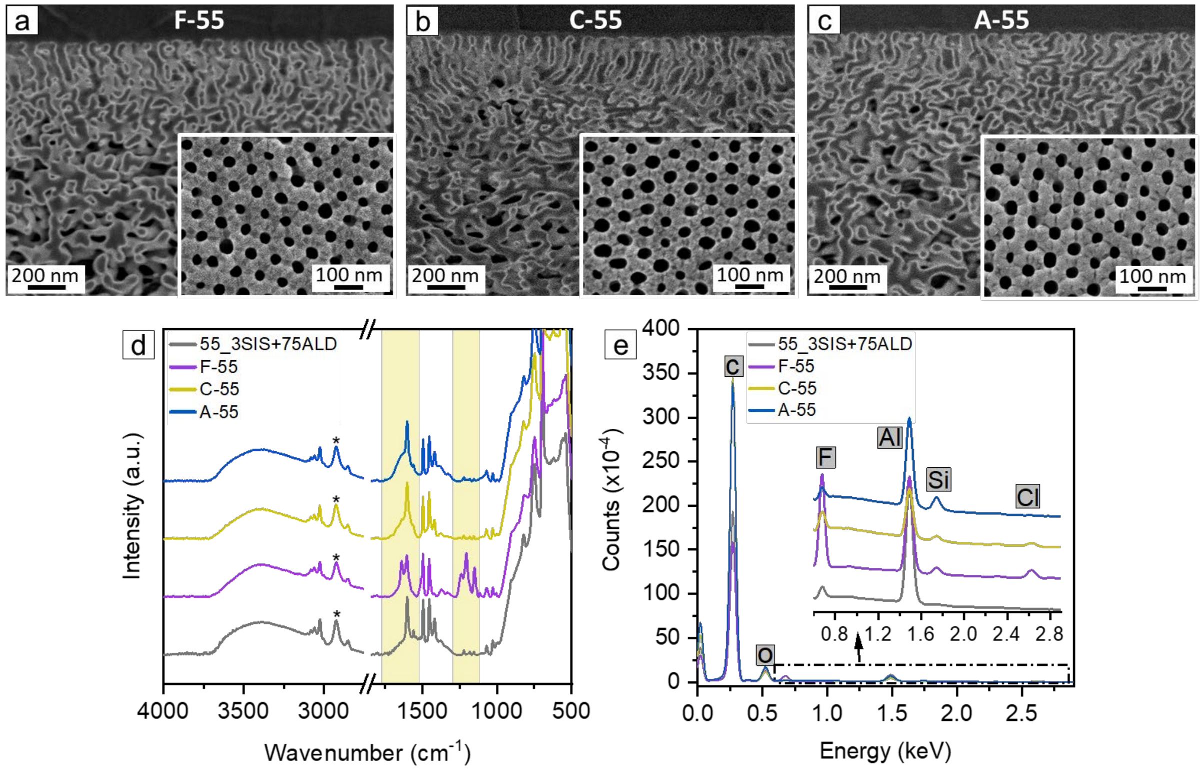

Figure S26. SEM images of (a) F-55 (fluorinated 55_3SIS+75ALD), (b) C-55 (cationic functionalized 55_3SIS+75ALD, (c) A-55 (anionic functionalized 55_3SIS+75ALD). (d) ATR-FTIR spectra of 55_3SIS+75ALD, F-55, C-55, A-55. The relative intensities were normalized using the characteristic CH_2 stretching vibration (*) of the unreactive PS-*b*-P4VP backbone around 2924 cm^{-1}. (e) Determination of the characteristic elements fluorine (F), silicon (Si) and chlorine (Cl) by EDX along the cross-section.

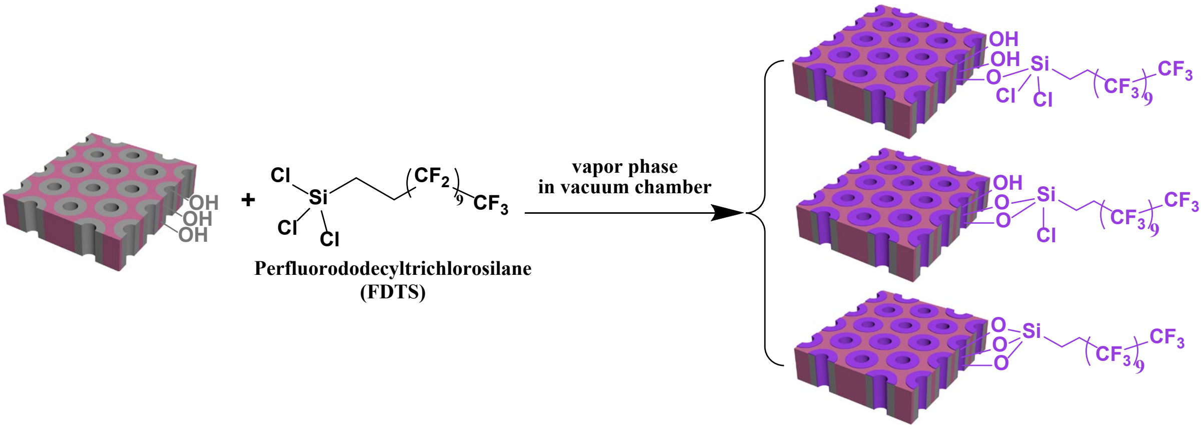

Figure S27. Schematic illustration of the reaction of perfluorododecyltrichlorosilane (FDTS) and hydroxyl groups on the 3SIS+75ALD modified membrane surface, e.g. 38_3SIS+75ALD and 55_3SIS+75ALD.

In addition to the strong signal of the characteristic element fluorine (F), EDX spectra of F-38 (Figure 4e in main manuscript) and F-55 (Figure S26e) exhibit the presence of the element chlorine (Cl). According to previous kinetic study of the reactions between chlorosilanes and the hydroxyl groups on silica, the multifunctional silanes (*i.e.*, di-, tri-, and tetrachlorosilanes) followed 1.5 ± 0.2-order kinetics with respect to the number of surface bonding sites.[3] Therefore, we expect that Si-Cl bonds of the multifunctional perfluorododecyltrichlorosilane (FDTS) did not completely react with hydroxyl groups on the AlO_x surface (Figure S27), as can be observed from the chlorine signal in the EDX spectrum.

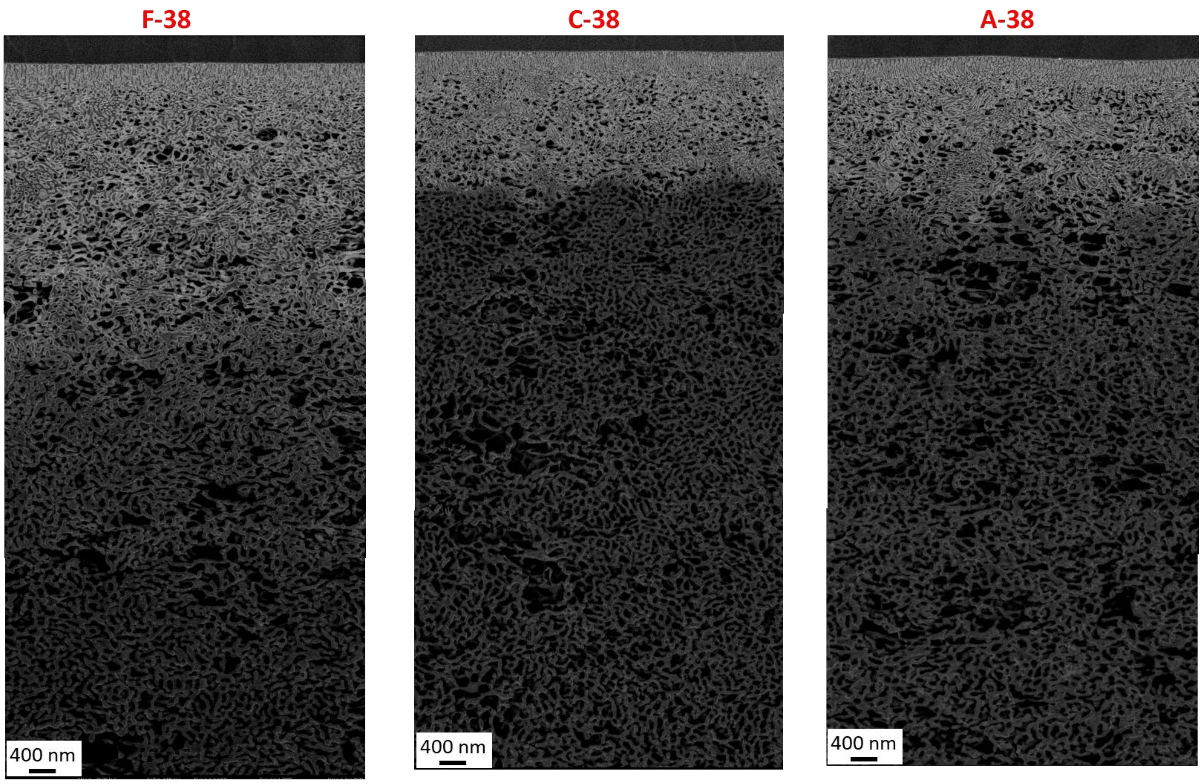

Figure S28. BSE images of the cross-section of the 38 nm series after silanization with low magnification.

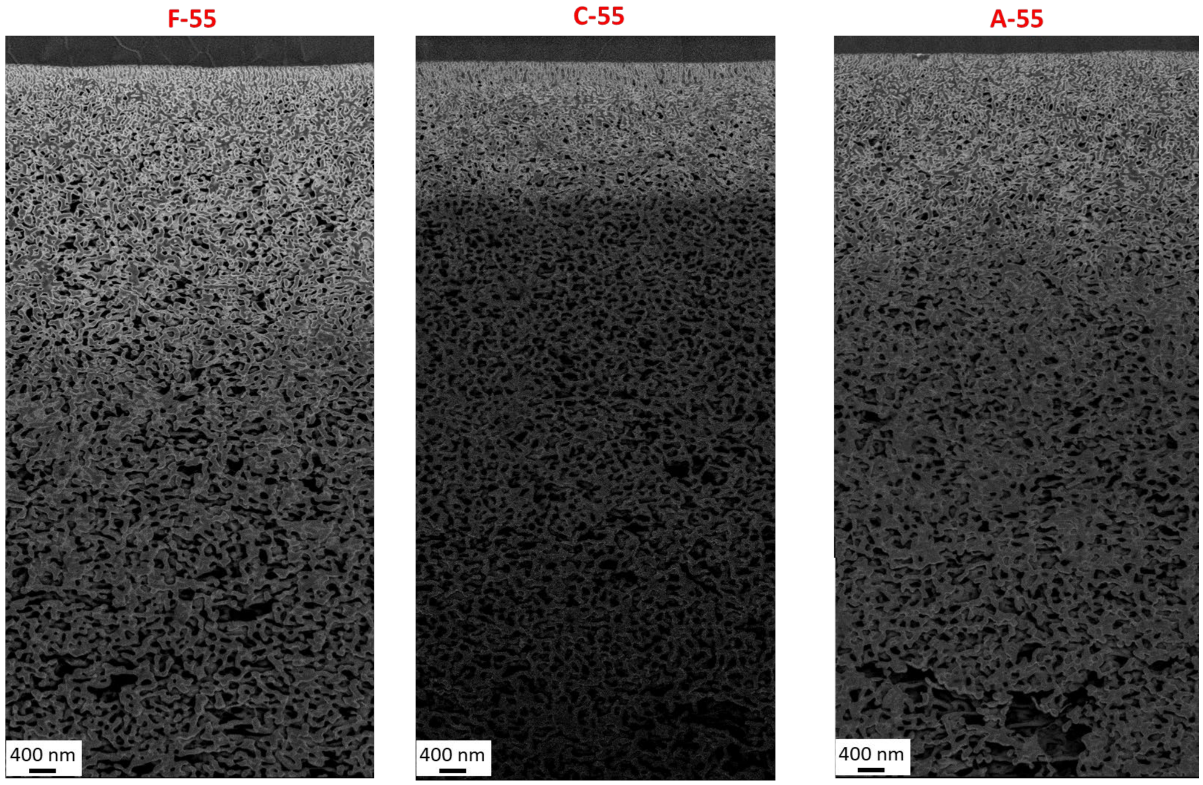

Figure S29. BSE images of the cross-section of the 55 nm series after silanization with low magnification.

Table S3. The average pore size of two series of membranes based on SEM images.

		Pri.	3SIS	3SIS+25ALD	3SIS+50ALD	3SIS+75ALD	Fluorinated 3SIS+75ALD	Cationic functionalized 3SIS+75ALD	Anionic functionalized 3SIS+75ALD
38 nm series	Pore size (nm)	37.8 ± 0.1	26.8 ± 1.1	24.0 ± 0.7	21.0 ± 0.6	18.5 ± 0.5	15.1 ± 0.9	17.5 ± 1.5	17.3 ± 0.4
	H_2O permeance ($Lm^{-2}h^{-1}bar^{-1}$)	1312 ± 39	1089 ± 113	982 ± 46	719 ± 44	522 ± 14	68 ± 3	492 ± 76	382 ± 8
55 nm series	Pore size (nm)	55.0 ± 0.6	42.1 ± 0.5	38.5 ± 0.5	35.6 ± 0.6	33.1 ± 0.4	27.4 ± 1.0	29.2 ± 0.6	29.1 ± 0.9
	H_2O permeance ($Lm^{-2}h^{-1}bar^{-1}$)	2232 ± 126	1936 ± 22	1742 ± 58	1557 ± 50	1058 ± 2	245 ± 47	784 ± 55	454 ± 30

Each average value of the pore size was calculated based on 3-6 SEM images.

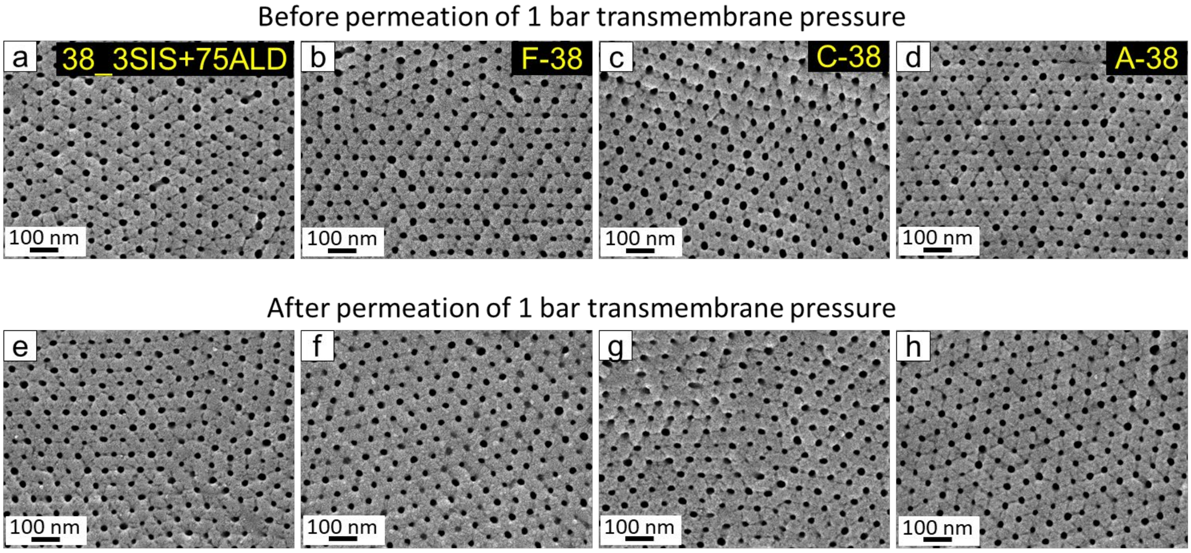

Figure S30. SEM images of the top surface of the membranes (a-d) before and (e-h) after permeation at a transmembrane pressure of 1 bar—(a, e) 38_3SIS+75ALD, (b, f) F-38, (c, g) C-38 and (d, h) A-38.

In order to investigate the stability of membrane isoporous structure during the pressure-driven permeation, we selected 38_3SIS+75ALD, F-38, C-38 and A-38 membranes as model systems, performed water permeance with a transmembrane pressure of 1 bar for 2h, and probed the membrane isoporous structure before and after permeation by SEM (Figure S30). SEM imaging shows that all membranes after permeation kept intact isoporous structure without structural change, indicating good mechanical robustness, in agreement with the previous study.[4]

2.3. Membrane performance

2.3.1. Membrane hydrophilicity and permeability

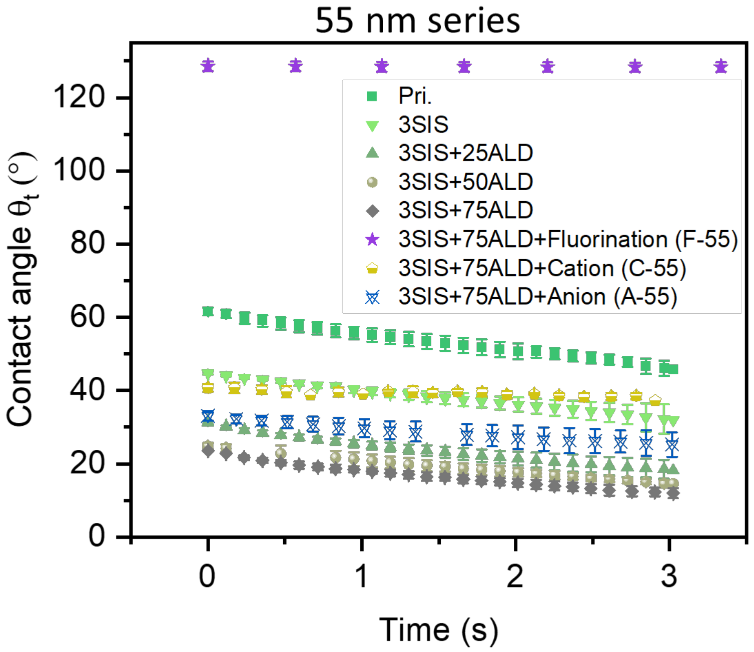

Figure S31. The change of the water contact angle on the membrane surface of the 55 nm series as a function of time.

The water contact angle θ of the two series of 38 nm and 55 nm membranes decreases with the increasing number of cycles of SIS and ALD treatment, following a sequence of $\theta_{Pri.} < \theta_{3SIS} < \theta_{3SIS+25ALD} < \theta_{3SIS+50ALD} < \theta_{3SIS+75ALD}$. 3SIS-treated membranes exhibit less hydrophilicity than the membranes with 3SIS plus additional ALD treatments, due to the exclusive growth of AlO_x layer within the P4VP pore-forming block, but not on the surface of the PS matrix block yet. Moreover, 3SIS+25ALD-treated membranes are slightly less hydrophilic than 3SIS+50ALD- and 3SIS+75ALD-treated membranes, implying a non-complete coverage of AlO_x layer on the surface of the PS matrix in case of merely 25 ALD cycles. Although we expected a complete coverage with AlO_x on the top surfaces of 3SIS+50ALD- and 3SIS+75ALD-treated membranes and consequently a similar hydrophilicity, the measured initial contact angles θ_0

had a very slight difference (Figure 5a in main manuscript and Figure S27). This likely can be attributed to the differences in the pore size and surface roughness instead of the hydrophilicity.

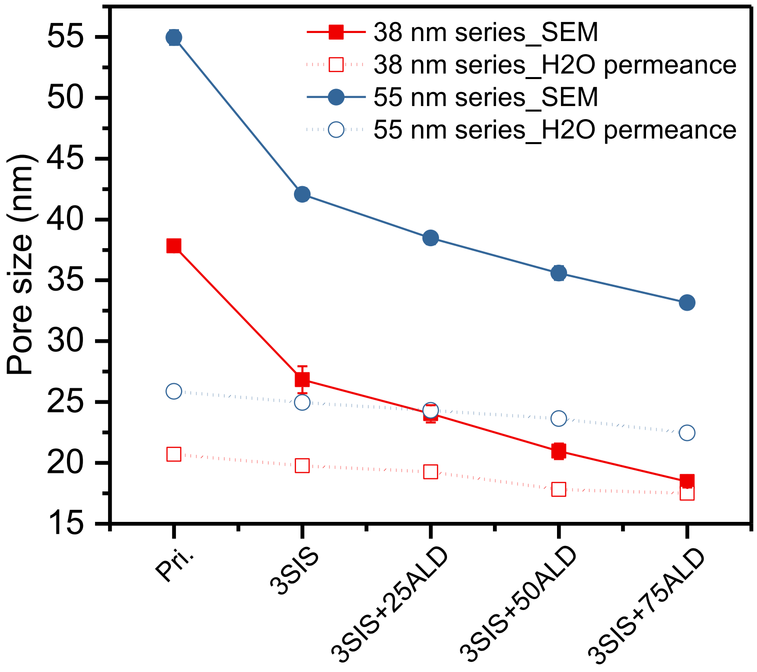

Figure S32. The comparison of pore sizes based on SEM images and calculated by the Hagen-Poiseuille equation based on water permeance.

Under the assumption that the resistance of the spongy substructure of the membrane is negligible and only the cylindrical ordered top layer of the membrane mainly hinders the permeance, the Hagen-Poiseuille equation can be used to calculate the effective pore size of membranes based on water flux J, as shown in the following Equation S5

$$J = N\frac{\pi d^4 \Delta p}{128\eta L} \tag{5}$$

Where N is the number of surface pores per unit area, d is the effective pore diameter, Δp is the transmembrane pressure, η is the water viscosity (8.94×10^{-4} Pa s at 24 °C) and L is the length of the cylindrical pores.

According to the top surface and cross-sectional SEM images of membranes, the 38 nm series had a pore number density N of 2.521×10^{14} m^{-2} and average length of cylindrical layer L of

350 nm, while the 55 nm series had $N = 1.505\times10^{14}$ m^{-2} and $L = 300$ nm. The employed transmembrane pressure Δp was 1 bar. The resulting effective pore sizes were compared with the pore size from SEM images in Figure S32. It is clear that the estimated values by the Hagen-Poiseuille equation are lower than those from SEM images. This discrepancy mainly arises from the rough assumption of negligible resistance of the spongy sublayer.[5] However, we observed that the gap between both methods is gradually reduced with more SIS and ALD cycles. This is because the Hagen-Poiseuille equation does not take into account the parameter of hydrophilicity, which means the contribution of the hydrophilicity of SIS and ALD-treated membranes to the water permeance is not negligible.[5b]

2.3.2. Membrane selectivity

Table S4. The molecular characteristics of the model organic molecules used for the molecular separation.

Name	M_w (g mol^{-1})	Structure	Net charge	pH in H_2O
Rose bengal (RB)	1017.64	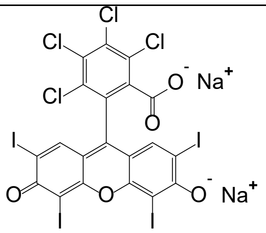	-2	6.72
Tris(bipyridine)ruthenium(II) chloride (Ru)	640.53	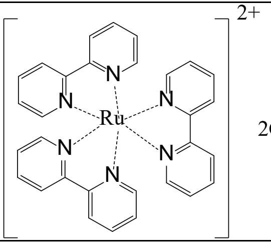	+2	6.59
Orange II (OR-)	350.32	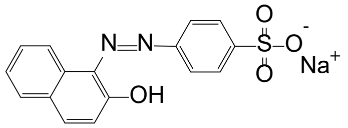	-1	6.7
Naphthol green B (NG3-)	878.46	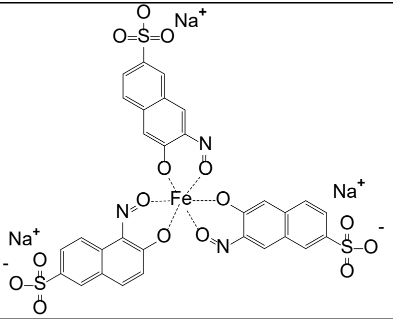	-3	6.4
Reactive green 19 (RG6-)	1418.93	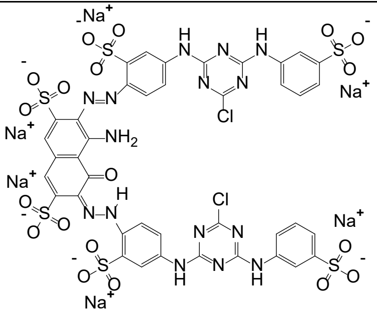	-6	4.82
β-Cyclodextrin (CD0)	1134.98	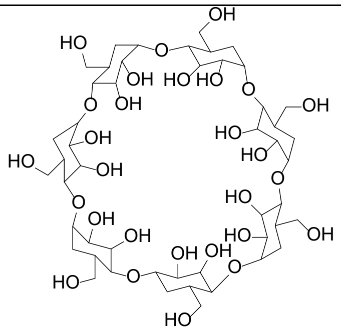	0	7.06

Mixture of OR- & RG6-	--	--	--	5.86

Table S5. The molecular characteristics of model proteins used for the molecular separation.

Name	M_w (kg mol^{-1})	Structure[a)]	Isoelectric point (IEP)	Net charge in H_2O	pH in H_2O
Lysozyme (LZ)	14.3	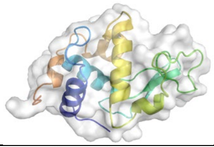	11.35[b)]	Positive	4.25
β-Lactoglobulin (LG)	18.4	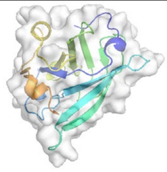	5.2-5.3[6]	Slightly negative	6.75

Note: [a)]Lysozyme PDB: 1DPX (structure of hen egg-white lysozyme); β-Lactoglobulin PDB: 1BEB (bovine beta-lactoglobulin, lacttice X); [b)]The isoelectric point (IEP) is provided by the supplier Sigma-Aldrich.

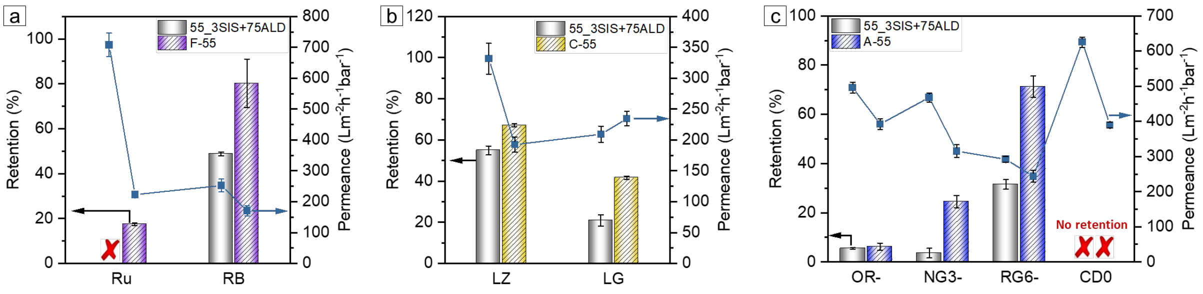

Figure S33. (a) Hydrophobicity-based, (b) charge-based and (c) size/charge-based selectivity between different organic solutes based on the fluorinated membrane F-55, cationic membranes 55_3SIS+75ALD and C-55, and anionic membrane A-55, respectively: retention (column, left Y-axis) and permeance (line+symbol, right Y-axis).

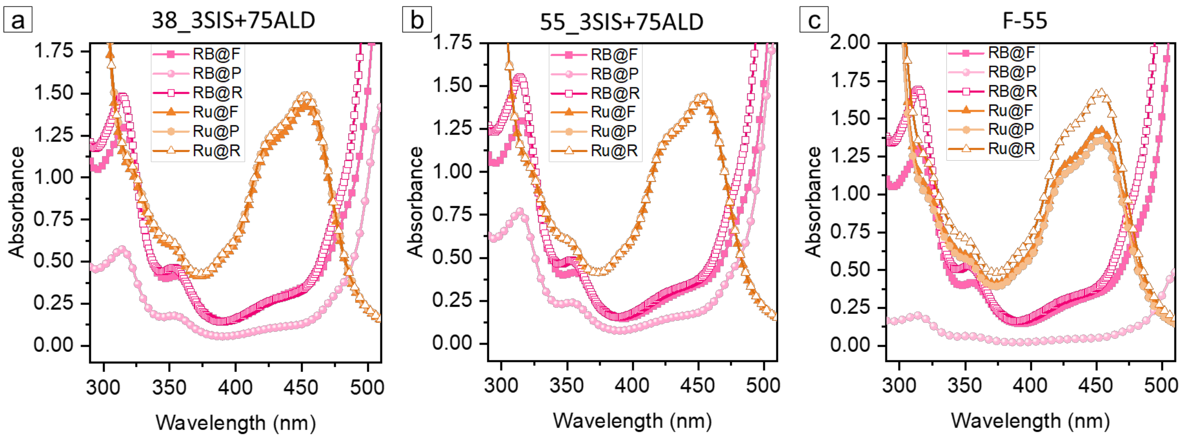

Figure S34. UV-vis spectra of the corresponding feed (F), permeate (P) and retentate (R) solutions obtained by (a) 38_3SIS+75ALD, (b) 55_3SIS+75ALD, (c) F-55.

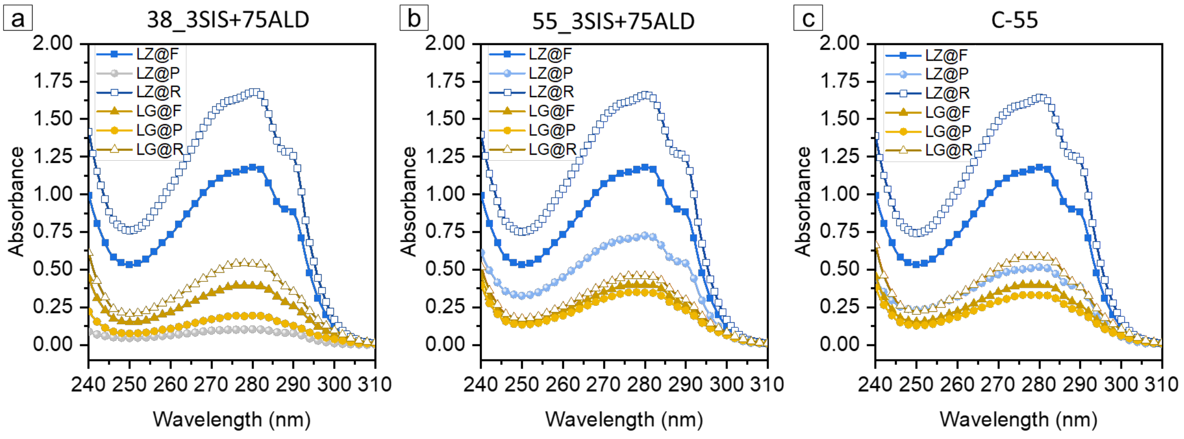

Figure S35. UV-vis spectra of the corresponding feed (F), permeate (P) and retentate (R) solutions obtained by (a) 38_3SIS+75ALD, (b) 55_3SIS+75ALD, (c) C-55.

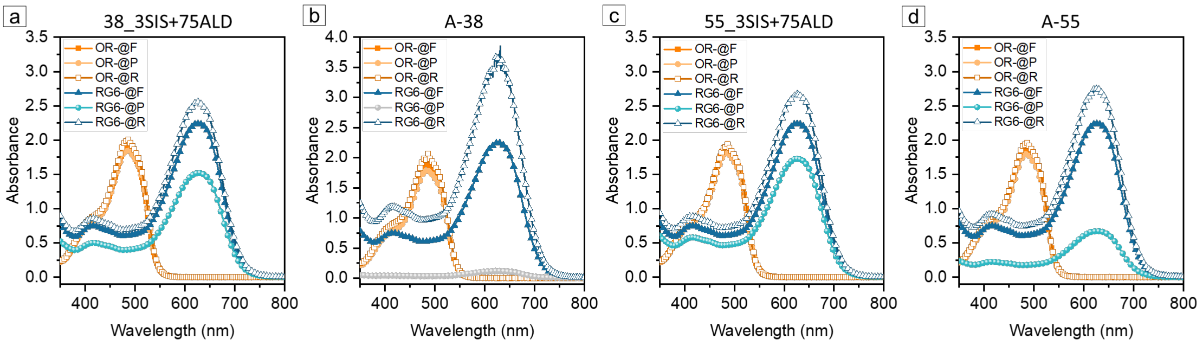

Figure S36. UV-vis spectra of the corresponding feed (F), permeate (P) and retentate (R) solutions obtained by (a) 38_3SIS+75ALD, (b) A-38, (c) 55_3SIS+75ALD, (d) A-55.

Table S6. The detailed values of retention, selectivity and permeance of Ru and RB for the membranes 38_3SIS+75ALD, F-38, 55_3SIS+75ALD and F-55.

Membrane	Retention (R, %)		Selectivity (ψ)	Permeance (P, L h^{-1} m^{-2} bar^{-1})	
	Ru	RB	$\Psi_{Ru/RB}$	Ru	RB
38_3SIS+75ALD	0	59.2 ± 1.1	2.5	424.7 ± 19.9	197.4 ± 4.7
F-38	31.5 ± 1.1	98.7 ± 0.9	52.7	25.3 ± 2.5	32.6 ± 4.1
55_3SIS+75ALD	0	48.8 ± 0.8	2.0	707.5 ± 38.3	252.6 ± 21.0
F-55	17.5 ± 0.6	80.2 ± 10.8	4.2	222.8 ± 9.1	170.3 ± 16.0

Table S7. The detailed values of retention, selectivity and permeance of LZ and LG for the membranes 38_3SIS+75ALD, C-38, 55_3SIS+75ALD and C-55.

Membrane	Retention (R, %)		Selectivity (ψ)	Permeance (P, L h^{-1} m^{-2} bar^{-1})	
	LZ	LG	$\Psi_{LG/LZ}$	LZ	LG
38_3SIS+75ALD	92.4 ± 0.8	63.6 ± 1.0	4.8	153.2 ± 11.5	141.9 ± 10.3
C-38	92.2 ± 1.1	57.1 ± 8.4	5.5	210.3 ± 11.3	138.7 ± 3.4
55_3SIS+75ALD	54.9 ± 2.1	20.9 ± 2.7	1.8	331.6 ± 24.9	209.3 ± 13.2
C-55	67.1 ± 0.8	41.7 ± 0.8	1.8	192.4 ± 12.4	234.4 ± 11.7

Table S8. The detailed values of retention, selectivity and permeance of OR-, NG3-, RG6- and CD0 for the membranes 38_3SIS+75ALD, A-38, 55_3SIS+75ALD and A-55.

Membrane	Retention (R, %)				Selectivity (ψ)			Permeance (P, L h^{-1} m^{-2} bar^{-1})			
	OR-	NG3-	RG6-	CD0	$\Psi_{OR-/RG6-}$	$\Psi_{NG3-/RG6-}$	$\Psi_{CD0/RG6-}$	OR-	NG3-	RG6-	CD0
38_3SIS+75ALD	8.2 ± 2.6	23.4 ± 4.7	40.6 ± 1.4	0	1.5	1.3	1.7	346.2 ± 24.3	313.7 ± 12.9	251.2 ± 1.8	402.3 ± 14.4
A-38	10.0 ± 0.8	26.9 ± 9.4	92.8 ± 0.6	0	12.5	10.2	13.9	333.8 ± 17.2	283.5 ± 4.1	244.5 ± 10.8	381.3 ± 19.3
55_3SIS+75ALD	5.4 ± 0.3	3.6 ± 2.0	31.5 ± 2.0	0	1.4	1.4	1.5	495.9 ± 15.9	468.1 ± 13.0	292.2 ± 8.2	625.4 ± 14.4
A-55	6.2 ± 1.5	24.5 ± 2.5	71.2 ± 4.4	0	3.3	2.6	3.5	392.3 ± 15.7	315.2 ± 18.1	243.4 ± 16.8	389.5 ± 8.6

Table S9. The detailed values of retention, selectivity and permeance of the mixture of OR- and RG6- for the membrane A-38.

Membrane	Retention (*R*, %)		Permeance (*P*, L h^{-1} m^{-2} bar^{-1})	Selectivity (ψ)
	OR-	RG6-		$\Psi_{OR-/RG6-}$
A-38	27.7 ± 9.3	93.8 ± 1.2	253.1 ± 14.0	11.7

Table S10. Performance comparison among various membranes for hydrophobicity, charge- and charge/size-based separation of small molecules.

Membrane type	Small molecules	Molecular weight (g mol^{-1})	Molecular charge	Selectivity_ diffusion[a)]	Selectivity_filtration	Permeance (Lm^{-2} h^{-1} bar^{-1})	Reference
Hydrophobicity-based separation							
Fluorinated PCTE membranes via iCVD	Mesitylene	120.19	0	37	--	--	[7]
	Phloroglucinol	126.11	0				
	4-Phenylazodiphenylamine	273.3	0	234			
	Alizarin Yellow GG	309.21	-1				
Fluorinated gold nanotube membranes	Pinacyanol chloride	388.94	+1	16.28	--	--	[8]
	Rose bengal	1017.64	-2				
NP-Den hybrid membrane	β -Naphthalene-sulfonic acid	208.23	-1	2.26	--	--	[9]
	para-toluene-sulfonic acid	172.2	-1				
Self-assembled polyelectrolyte deposited PCTE	p-nitrotoluene	137.14	0	1.3	--	--	[10]
	p-nitrophenol	139.11	0				
Fluorinated SiO_2/AAO membrane	Tris(bipyridine)ruthenium(II) chloride	640.53	+2	5.52	--	--	[11]
	Rose bengal	1017.64	-2				
C-38	Tris(bipyridine)ruthenium(II) chloride	640.53	+2	--	52.7	25.3	This work
	Rose bengal	1017.64	-2			32.6	

Charge- and charge/size-based separation								
NP-Den hybrid membrane	Rhodamine 6G	479.02	+1	11	--		--	[9]
	Calcein	622.53	-4					
Self-assembled polyelectrolyte deposited PCTE	Rhodamine 6G	479.02	+1	3.5	--		--	[10]
	Calcein	622.53	-4					
Cationic dendrimer deposited PCTE	Rhodamine 6G	479.02	+1	10	--		--	[12]
	Calcein	622.53	-4					
					Single solutes	Mixed solutes		
Amphiphilic random copolymer membrane	Riboflavin	376.36	0	263	8.4 b)	19.2 b)	4.2 c)	[13]
	Acid blue 45	474.33	-2					
MM	Riboflavin	376.36	0	--	21.3	28.3	11.0 c)	[2a]
	Methylene blue	319.85	+1					
PM	Orange II	350.32	-1	--	14.7	44.6	9.5 c)	
	Naphthol green B	878.45	-3					
	Orange II	350.32	-1	--	64.3	--		
	Reactive green 19	1418.93	-6					
SM	Orange II	350.32	-1	--	5.2	--	60	[2b]
	Reactive green 19	1418.93	-6					
A-38	Orange II	350.32	-1	--	12.5	11.7	253.1 d)	This work
	Reactive green 19	1418.93	-6					
	Naphthol green B	878.45	-3	--	10.2	--	283.5	
	Reactive green 19	1418.93	-6				244.5	
	β-Cyclodextrin	1134.98	0	--	13.9	--	381.3	
	Reactive green 19	1418.93	-6				244.5	

a)The selectivity based on the diffusion test determined using a single solute system. b)The selectivities are calculated by us using the reported retention values. c)The value is ultrapure water permeance. d)The value is the permeance of the mixed solute solution. PCTE: polycarbonate track-etched membrane.

References

[1] S. Rangou, K. Buhr, V. Filiz, J. I. Clodt, B. Lademann, J. Hahn, A. Jung, V. Abetz, *Journal of Membrane Science* **2014**, 451, 266.

[2] a) Z. Zhang, M. M. Rahman, C. Abetz, A. L. Höhme, E. Sperling, V. Abetz, *Adv. Mater.* **2020**, 32, 1907014; b) Z. Zhang, M. M. Rahman, C. Abetz, V. Abetz, *J. Mater. Chem. A* **2020**, 8, 9554.

[3] M. L. Hair, W. Hertl, *The Journal of Physical Chemistry* **1969**, 73, 2372.

[4] J. I. Clodt, B. Bajer, K. Buhr, J. Hahn, V. Filiz, V. Abetz, *Journal of Membrane Science* **2015**, 495, 334.
[5] a) K. V. Peinemann, V. Abetz, P. F. Simon, *Nat. Mater.* **2007**, 6, 992; b) Z. Zhang, M. M. Rahman, C. Abetz, B. Bajer, J. Wang, V. Abetz, *Macromol. Rapid Commun.* **2019**, 40, 1800729.
[6] a) D. Mercadante, L. D. Melton, G. E. Norris, T. S. Loo, M. A. Williams, R. C. Dobson, G. B. Jameson, *Biophys. J.* **2012**, 103, 303; b) Y. Yan, D. Seeman, B. Zheng, E. Kizilay, Y. Xu, P. L. Dubin, *Langmuir* **2013**, 29, 4584.
[7] A. Asatekin, K. K. Gleason, *Nano Lett.* **2011**, 11, 677.
[8] L. Velleman, J. G. Shapter, D. Losic, *Journal of Membrane Science* **2009**, 328, 121.
[9] M. H. Park, C. Subramani, S. Rana, V. M. Rotello, *Adv. Mater.* **2012**, 24, 5862.
[10] E. N. Savariar, K. Krishnamoorthy, S. Thayumanavan, *Nat. Nanotechnol.* **2008**, 3, 112.
[11] L. Velleman, G. Triani, P. J. Evans, J. G. Shapter, D. Losic, *Microporous Mesoporous Mater.* **2009**, 126, 87.
[12] E. N. Savariar, M. M. Sochat, A. Klaikherd, S. Thayumanavan, *Angew. Chem. Int. Ed. Engl.* **2009**, 48, 110.
[13] I. Sadeghi, J. Kronenberg, A. Asatekin, *ACS Nano* **2018**, 12, 95.
